# Transcriptional co-factor Transducin beta-like (TBL) 1 acts as a checkpoint in pancreatic cancer malignancy

**DOI:** 10.15252/emmm.201404837

**Published:** 2015-06-13

**Authors:** Christian Stoy, Aishwarya Sundaram, Marcos Rios Garcia, Xiaoyue Wang, Oksana Seibert, Annika Zota, Susann Wendler, David Männle, Ulf Hinz, Carsten Sticht, Maria Muciek, Norbert Gretz, Adam J Rose, Vera Greiner, Thomas G Hofmann, Andrea Bauer, Jörg Hoheisel, Mauricio Berriel Diaz, Matthias M Gaida, Jens Werner, Tobias Schafmeier, Oliver Strobel, Stephan Herzig

**Affiliations:** 1 Joint Division Molecular Metabolic Control, German Cancer Research Center (DKFZ) Heidelberg, Center for Molecular Biology (ZMBH) and University Hospital, Heidelberg UniversityHeidelberg, Germany; 2 Institute for Diabetes and Cancer IDC, Helmholtz Center Munich and Joint Heidelberg-IDC Translational Diabetes ProgramNeuherberg, Germany; 3 Department of General Surgery, University Hospital HeidelbergHeidelberg, Germany; 4 Medical Research Center, Klinikum MannheimMannheim, Germany; 5 Research Group Cellular Senescence, German Cancer Research Center (DKFZ) HeidelbergHeidelberg, Germany; 6 Functional Genome Analysis, German Cancer Research Center (DKFZ) HeidelbergHeidelberg, Germany; 7 Institute of Pathology, Heidelberg UniversityHeidelberg, Germany

**Keywords:** gemcitabine, pancreatic cancer, PI3 kinase, TBL1, tumor metabolism

## Abstract

Pancreatic ductal adenocarcinoma (PDAC) is the fourth leading cause of cancer fatalities in Western societies, characterized by high metastatic potential and resistance to chemotherapy. Critical molecular mechanisms of these phenotypical features still remain unknown, thus hampering the development of effective prognostic and therapeutic measures in PDAC. Here, we show that transcriptional co-factor Transducin beta-like (TBL) 1 was over-expressed in both human and murine PDAC. Inactivation of TBL1 in human and mouse pancreatic cancer cells reduced cellular proliferation and invasiveness, correlating with diminished glucose uptake, glycolytic flux, and oncogenic PI3 kinase signaling which in turn could rescue TBL1 deficiency-dependent phenotypes. TBL1 deficiency both prevented and reversed pancreatic tumor growth, mediated transcriptional PI3 kinase inhibition, and increased chemosensitivity of PDAC cells *in vivo*. As TBL1 mRNA levels were also found to correlate with PI3 kinase levels and overall survival in a cohort of human PDAC patients, TBL1 was identified as a checkpoint in the malignant behavior of pancreatic cancer and its expression may serve as a novel molecular target in the treatment of human PDAC.

## Introduction

Malignancy of the exocrine pancreas, in particular ductal adenocarcinoma (PDAC), is a detrimental disease with high lethality and limited therapeutic options. This severe form of cancer causes more than 200,000 deaths worldwide every year and has a 5-year survival rate of only 4%. It is therewith the fourth leading cause of cancer fatalities in Western societies (Raimondi *et al*, 2009; Vincent *et al*, 2011). At diagnosis, PDAC is already metastasized or locally advanced and non-resectable in more than 80% of cases and only poorly responds to chemotherapeutic treatment (Vincent *et al*, 2011).

An early event in PDAC tumorigenesis is the metaplastic reorganization of acinar cells into duct-like structures (ADM), which commonly occurs in response to inflammatory challenges (Reichert & Rustgi, 2011). Metaplasia has been at least partially attributed to acinar-to-ductal transdifferentiation in the adult pancreas (Strobel *et al*, 2007). Initial metaplasia might progress into cancer precursor lesions referred to as pancreatic intraepithelial neoplasia (PanIN) (Vincent *et al*, 2011). A variety of oncogenic pathways has been identified in murine and human PDAC, most notably activating mutations of the KRAS proto-oncogene, which has been accounted for as the main driver of PDAC tumor formation and progression (Hidalgo, 2010; Vincent *et al*, 2011). Oncogenic KRAS in turn triggers a number of downstream signaling pathways, including Raf/MEK/ERK, RalGDS/p38 MAPK, Rac and Rho, and phosphatidylinositide 3 (PI3) kinase (Castellano & Downward, 2011), the latter of which seems to be responsible for the majority of KRAS-dependent pancreatic tumorigenesis (Eser *et al*, 2013). Yet, the detailed molecular mechanisms underlying PDAC oncogenesis as well as those determining the remarkable and clinically detrimental therapy resistance are still poorly understood, thereby hampering the development of effective therapeutic measures.

By wiring distinct transcriptional networks through gene-specific interactions with DNA-binding transcription factors, transcriptional co-factor complexes have been identified as critical checkpoints in the coordination of distinct cellular programs (Spiegelman & Heinrich, 2004). In this respect, particularly the differential gene expression of specific transcriptional regulators between healthy and malignant conditions has often been found to reflect a causal, functional role of these factors distinct physiological and pathophysiological settings (Berriel Diaz *et al*, 2008; Jones *et al*, 2013).

Based on the multi-level cellular dysfunction in pancreatic cancer, we hypothesized that the aberrant expression of as-yet-not-identified transcriptional co-factor complexes during PDAC development directly mirrors the tumorigenic status of pancreatic tumor cells and may contribute to the progression and the adverse outcome of this disease. Transducin beta-like 1 (TBL1) represents a transcriptional co-factor that has been initially described as a component of the nuclear receptor co-repressor (NCoR)/silencing mediator for retinoid and thyroid receptors (SMRT)/histone deacetylase (HDAC) 3 complex (Guenther *et al*, 2000). Most recently, we have defined the aberrant expression of TBL1/TBLR1 as a central event of pathophysiological lipid homeostasis in metabolically active tissues (Kulozik *et al*, 2011; Rohm *et al*, 2013). Of note, TBL1 has also been linked to the oncogenic Wnt signaling pathway (Li & Wang, 2008).

Given the tight association between PDAC and metabolic dysfunction (Giovannucci & Michaud, 2007), we thus tested the hypothesis that the TBL1 transcriptional complex may represent an integrator of proliferative and metabolic pathways in pancreatic cancer.

## Results

### TBL1 is highly over-expressed in human PDAC

**Figure 1:**
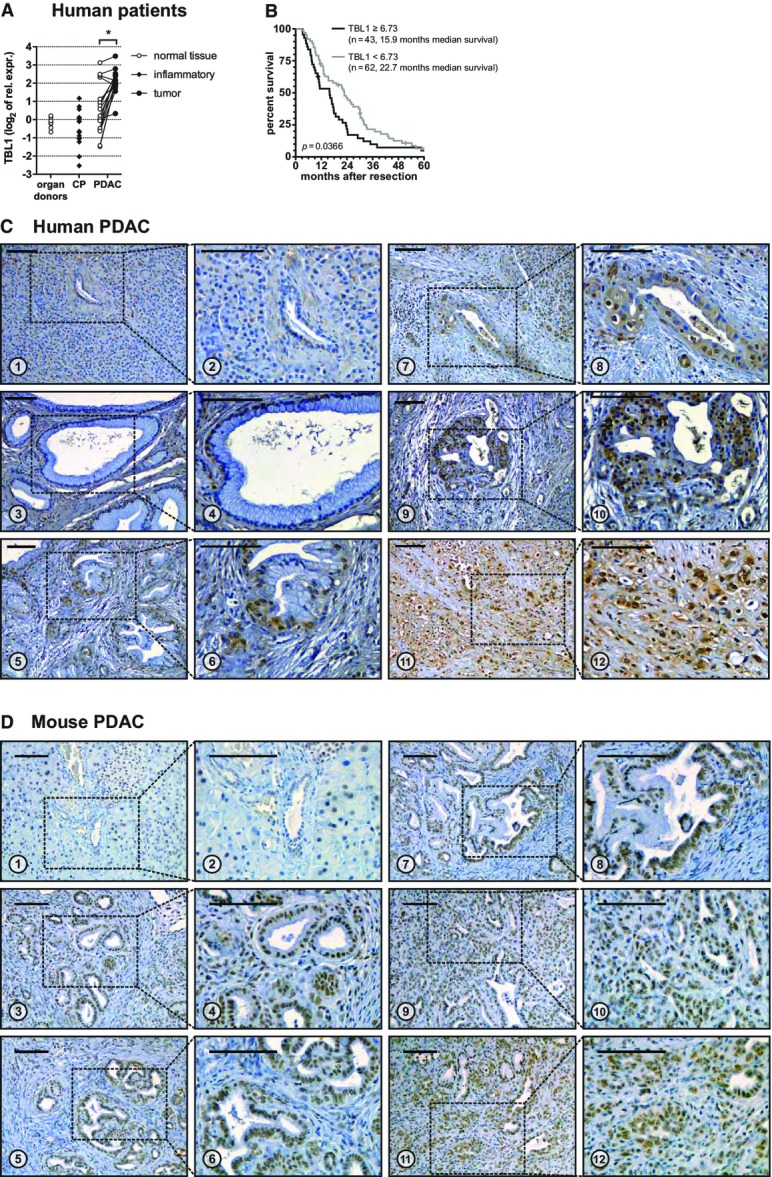
TBL1 is overexpressed in human and mouse PDAC mRNA expression of individual patients normalized to 18S-rRNA and relative to a pooled organ donor sample. Lines between dots connect samples from the same patient. CP: chronic pancreatitis; PDAC: pancreatic ductal adenocarcinoma; significant difference (*P* < 0.05) between tumor and normal tissue *P* = 9.39 × 10^−5^ determined by paired two-tailed Student’s *t*-test, *n *=* *17 patients.

To initially explore a potential involvement of TBL1 in PDAC, we determined the expression levels of TBL1 in the human pancreas from patients who had undergone surgery for PDAC (Table1). Samples obtained from organ donors were used as reference. TBL1 mRNA levels were detectable in tumor-free pancreatic tissue, albeit at a relatively low level. In contrast, pancreatic cancer tissue from the same patients displayed high TBL1 mRNA levels (Fig1A), suggesting that differential expression of TBL1 is associated with tumorigenesis and/or staging in humans. Notably, we could neither observe an influence of body mass index (BMI) (< 25 vs. > 30 kg/m^2^) nor of chronic pancreatitis on TBL1 levels, indicating that systemic metabolic dysfunction and an inflammatory state are not causative for TBL1 induction. Subsequent histological examination demonstrated that high levels of TBL1 protein were restricted to neoplastic epithelial cells in both cytoplasmic as well as nuclear compartments but could hardly be detected in other cell types within the pancreas, and gradually increased from non-malignant tissue to PanIN lesions and invasive adenocarcinoma (Fig1C; Supplementary Fig S1).

**Table 1:** Patient statistics

	Organ donors	Chronic pancreatitis	PDAC, BMI ≤ 25	PDAC, BMI ≥ 30
*n*	8	15	9	9
Mean age ± SD	44.25 ± 21.45	49.50 ± 12.52	63.89 ± 12.42	68.44 ± 7.88
Male	6/8 (75%)	10/15 (66.7%)	4/9 (44.4%)	4/9 (44.4%)
Female	2/8 (25%)	5/15 (33.3%)	5/9 (55.6%)	5/9 (55.6%)
Mild pancreatitis	n.a.	2/15 (13.3%)	n.a.	n.a.
Medium-severe pancreatitis	n.a.	4/15 (26.7%)	n.a.	n.a.
Severe pancreatitis	n.a.	9/15 (60%)	n.a.	n.a.
Mean BMI ± SD	n.d.	n.d.	23.04 ± 1.42	32.33 ± 1.52
T3N0M0	n.a.	n.a.	2/9 (22.2%)	0/9 (0%)
T3N1M0	n.a.	n.a.	6/9 (66.7%)	7/9 (77.8%)
T3N1M1	n.a.	n.a.	1/9 (11.1%)	2/9 (22.2%)

Statistical parameters of organ donors, chronic pancreatitis, and PDAC patients used for the initial screen shown in Figs1A–C and 3D and E. *n*: total number of patients, SD: standard deviation, n.a.: not applicable, n.d.: not determined.

Association of microarray-based TBL1 expression and survival in 105 patients with PDAC: High TBL1 expression is significantly associated with shorter post-resection survival. *P*-value determined by log-rank test as described in Materials and Methods.

Immunohistochemistry staining for TBL1 in human paraffin-embedded tissue sections. 1, 2: healthy tissue with acini and duct showing no or only very weak staining. 3, 4: PanIN-1 lesions with occasional nuclear and cytoplasmic staining. 5, 6: PanIN-2 lesion with nuclear and occasional cytoplasmic staining. 7–10: PanIN-3 lesion with nuclear and increased occurrence and intensity of cytoplasmatic staining. 11, 12: invasive pancreatic ductal adenocarcinoma cells with nuclear and cytoplasmic staining. Scale bars: 100 μm. Images are representative of 12 patient samples.

Immunohistochemistry staining for TBL1 in paraffin-embedded tissue sections of wild-type or p48^+/Cre^; Kras^+/^^LSL^^-G12D^ mice. 1, 2: healthy tissue with acini, islet of Langerhans, and duct (zoom-in in panel 2) showing occasional weak nuclear staining. 3, 4: PanIN-1 lesions with nuclear staining. 5, 6: PanIN-2 lesions with nuclear staining. 7, 8: PanIN-3 lesion with nuclear staining. 9–12: invasive pancreatic ductal adenocarcinoma cells with nuclear and occasional faint cytoplasmic staining. Scale bars: 100 μm. Images are representative of 32 wild-type and 29 p48^+/Cre^; Kras^+/^^LSL^^-G12D^ mice.

To validate these findings and to assess the potential clinical relevance of TBL1 in PDAC, we analyzed TBL1 expression in PDAC tissue with respect to overall survival in an independent cohort of 105 patients who underwent resection for PDAC. Intriguingly, based on genome-wide transcriptome analysis and after adjustment for effects of variable acinar cell content in pancreatic biopsies of these patients, TBL1 expression was significantly associated with post-resection overall survival in these subjects (Fig1B).

To test whether TBL1 upregulation is a conserved feature between human and mouse PDAC, we employed p48^+/Cre^; Kras^+/LSL-G12D^ mice. These animals develop pre-cancerous lesions and eventually malignant tumors in the pancreas due to organ-specific expression of a constitutively active KRAS small GTPase, thereby closely mimicking the human pathology (Hingorani *et al*, 2003). Similar to our observations in humans, TBL1 was found to be highly expressed in pancreatic tissue of KRAS mutant animals with particular enrichment in mouse PanIN lesions (Fig1D; Supplementary Fig S2), overall suggesting that the up-regulation of TBL1 represents a conserved component of the tumorigenic program in PDAC.

### TBL1 controls cellular tumorigenic and metabolic adaptations of pancreatic cancer cells

**Figure 2:**
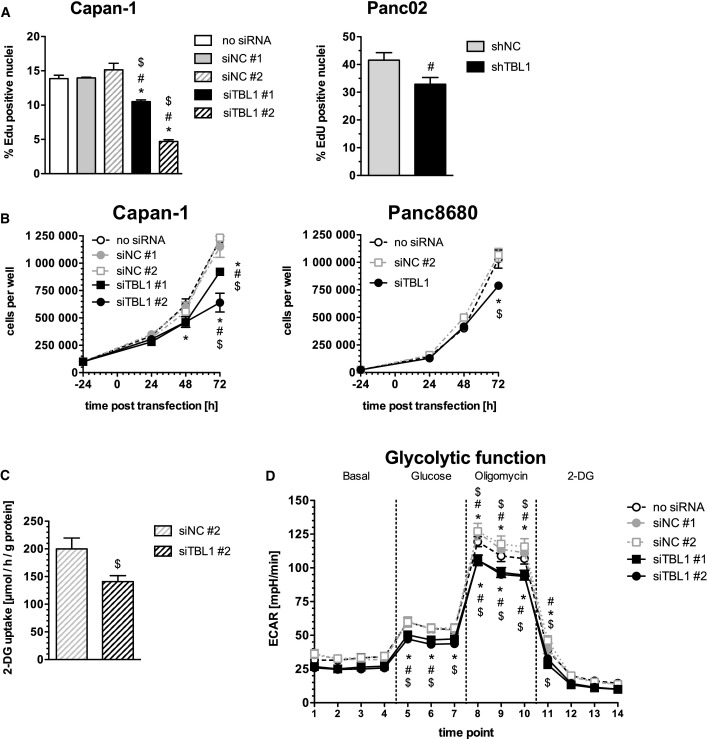
TBL1 controls pancreatic cancer cell growth and metabolic adaptations Left panel: EdU incorporation in siRNA-transfected Capan-1 cells (72 h post-transfection), *n *=* *6 cell culture wells per group; significantly different (*P* < 0.05) from control (no siRNA) *, siNC #1 #, siNC #2 $; *P*-values: siTBL1#1 8.24 × 10^−4^ (*), 5.43 × 10^−4^ (#), 7.93 × 10^−6^ ($); siTBL1 #2 1.66 × 10^−11^ (*), 1.27 × 10^−11^ (#), 8.79 × 10^−13^ ($), one-way ANOVA with Bonferroni post-test. Right panel: EdU incorporation of Panc02 cells with stable expression of shRNA; *n *=* *6 cell culture wells per group; significantly different (*P* < 0.05) from control (shNC) ^#^*P* = 0.0398; Welch’s *t*-test.

The marked upregulation of TBL1 expression in invasive PDAC next prompted us to explore the functional importance of TBL1 for pancreatic cancer cell properties. siRNA-mediated knockdown of TBL1 in human pancreatic ductal adenocarcinoma (Capan-1) cells (Supplementary Fig S3A, C and D) led to a significant reduction in cell proliferation and cell numbers compared with control siRNA-transfected cells as demonstrated by EdU incorporation and cell counting (Fig2A and B). Importantly, siRNA- or shRNA-mediated knockdown of TBL1 in murine pancreatic cancer cells (Panc02 and Panc8680) also reduced cell proliferation (Fig2A and B; Supplementary Fig S3B), underlining the conclusion that TBL1 fulfills a conserved growth regulatory function in both human and murine pancreatic cancer cells. Of note, knockdown of TBL1 in both human and murine pancreatic cancer cells significantly impaired tumor cell invasiveness (Supplementary Fig S4), overall suggesting that TBL1 represents a checkpoint in the tumorigenic program of pancreatic tumor cells.

Cell growth of siRNA-transfected Capan-1 or Panc8680 cells; *n *=* *3 cell culture wells per group; significantly different (*P* < 0.05) from control (no siRNA) *, siNC #1 #, siNC #2 $; *P*-values: Capan-1 cells 48 h time-point: 0.0408 (*) for siTBL1 #1 / 0.0464 for siTBL1 #2, Capan-1 cells 72 h time-point: 8.60 × 10^−5^ (*), 1.82 × 10^−3^ (#), 3.24 × 10^−5^ ($) for siTBL1 #1 / 2.39 × 10^−10^ (*), 3.34 × 10^−9^ (#), 1.08 × 10^−10^ ($) for siTBL1 #2; Panc8680 72 h time-point: 1.55 × 10^−4^ (*), 2.73 × 10^−5^ ($); two-way ANOVA with Bonferroni post-test.

Uptake of radio-labeled ^3^H-2-deoxyglucose in siRNA-transfected Capan-1 cells (48 h post-transfection); *n *=* *6 cell culture wells per group; statistically different (*P* < 0.05) from control (siNC #2) ^$^*P* = 0.03; Welch’s *t*-test.

Extracellular flux measurement of siRNA-transfected Capan-1 cells with glycolysis stress test assay (48 h post-transfection); 2-DG: 2-deoxyglucose; ECAR: extracellular acidification rate. *n *=* *6–8 cell culture wells per group; significantly different (*P* < 0.05) from control (no siRNA) *, siNC #1 #, siNC #2) $. Upper symbols: statistics for siTBL#1, *P*-values: 2.18 × 10^−3^ (*) time-point 8, 9.83 × 10^−3^ (*) time-point 9, 0.0102 (*) time-point 10, 0.044 (*) time-point 11; 2.42 × 10^−6^ (#) time-point 8, 1.47 × 10^−4^ (#) time-point 9, 3.17 × 10^−4^ (#) time-point 10, 0.0277 (#) time-point 11; 9.44 × 10^−7^ ($) time-point 8, 1.23 × 10^−6^ ($) time-point 9, 1.33 × 10^−6^ ($) time-point 10, 7.14 × 10^−5^ ($) time-point 11. Lower symbols: statistics for siTBL#2, *P*-values: 3.94 × 10^−3^ (*) time-point 5, 0.0143 (*) time-point 6, 0.0342 (*) time-point 7, 1.45 × 10^−3^ (*) time-point 8, 1.27 × 10^−3^ (*) time-point 9, 2.38 × 10^−3^ (*) time-point 10; 0.01 (#) time-point 5, 0.0362 (#) time-point 6, 1.22 × 10^−6^ (#) time-point 8, 1.28 × 10^−5^ (#) time-point 9, 5.84 × 10^−5^ (#) time-point 10; 0.0118 ($) time-point 5, 0.0208 ($) time-point 6, 0.0291 ($) time-point 7, 4.52 × 10^−7^ ($) time-point 8, 6.04 × 10^−8^ ($) time-point 9, 1.43 × 10^−7^ ($) time-point 10, 4.83 × 10^−3^ ($) time-point 11. Two-way ANOVA with Bonferroni post-test. Data information: All data plotted as mean ± SEM.

Rapidly proliferating cancer cells display distinct nutrient liabilities (Ward & Thompson, 2012), which prompted us to also test for potential metabolic adaptations in response to TBL1 deficiency in pancreatic cancer cells. Inactivation of TBL1 by siRNA delivery into Capan-1 cells reduced the cellular glucose uptake by 30% as measured 48 h after transfection by monitoring incorporation of radio-labeled 2-deoxyglucose over 15 min (Fig2C). These experiments suggested that TBL1 may couple proliferative, invasive, and metabolic pathways in pancreatic tumor cells. In agreement with a reduced cellular glucose supply upon TBL1 deficiency, TBL1-depleted Capan-1 cells diminished glycolytic glucose usage, while mitochondrial oxidative phosphorylation and mitochondrial ATP production remained unchanged as determined by metabolic flux analysis (Fig2D; Supplementary Fig S5).

### TBL1 is a direct upstream transcriptional activator of the PI3 kinase-dependent signaling pathway

**Figure 3:**
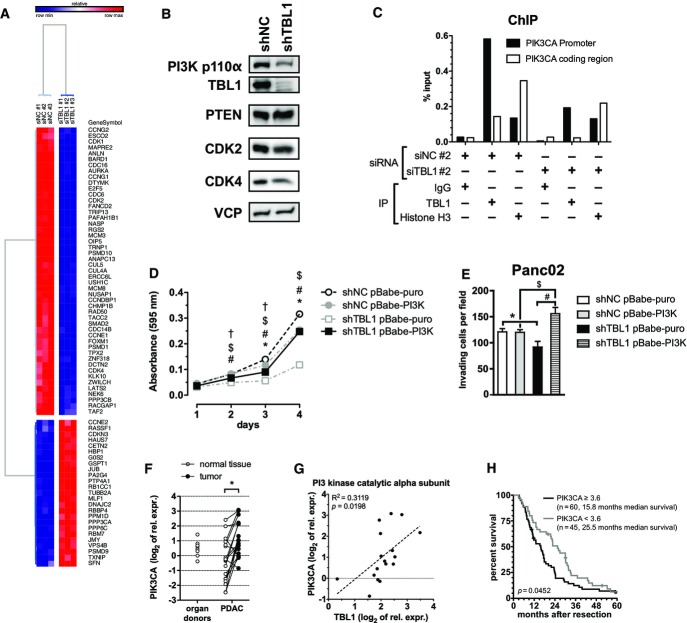
TBL1 controls PI3 kinase signaling by direct transcriptional regulation Gene expression microarray from Capan-1 cells transfected with siRNA. Heatmap shows genes annotated to KEGG pathway hsa04110 “Cell Cycle” and/or gene ontology cluster GO:0007049 “Cell Cycle”.

In order to next determine the molecular mechanisms of TBL1 action in pancreatic tumor cells, we performed high-throughput transcriptome analysis using Capan-1 cells. Genetic pathway determination revealed that pathways involved in cell proliferation and/or cell cycle were over-represented among those differentially regulated by TBL1 deficiency in these cells (Fig3A, Table2). Indeed, CDK2 and CDK4, which are important for G1/S-phase transition and G1 phase progression, respectively, were reduced following ablation of TBL1 in human and mouse cells (Figs3B and 5; Supplementary Fig S6A and B). In line with these findings, FACS analysis indeed demonstrated a decrease in the number of cells in S-phase and a concomitant increase in G0/G1 upon TBL1 knockdown as compared with control cells (Supplementary Fig S7). Of note, no major change in metabolic gene pathways was observed in response to TBL1 inhibition (Supplementary Table S1), implying that the metabolic alterations described above (Fig2C and D) may also represent secondary consequences of alterations in proliferation and/or cell growth.

**Table 2:** Over-representation analysis for TBL1 knockdown on GOPB terms

Annotation cluster 1		Enrichment score: 4.0244									
Term		Count	%	*P*-value	List total	Pop hits	Pop total	Fold enrichment	Bonferroni	Benjamini	FDR [%]
GO:0007049	Cell cycle	71	8.17	8.05E-08	635	776	13,528	1.949	2.18E-04	2.18E-04	1.43E-04
GO:0000278	Mitotic cell cycle	39	4.49	4.61E-06	635	370	13,528	2.246	0.012382	0.006210	0.008198
GO:0022403	Cell cycle phase	42	4.83	4.82E-06	635	414	13,528	2.161	0.012942	0.004333	0.008571
GO:0022402	Cell cycle process	52	5.98	5.15E-06	635	565	13,528	1.961	0.013830	0.003476	0.009163
GO:0051329	Interphase of mitotic cell cycle	16	1.84	8.65E-05	635	103	13,528	3.309	0.208732	0.025678	0.153934
GO:0051325	Interphase	16	1.84	1.21E-04	635	106	13,528	3.216	0.278569	0.032125	0.214621
GO:0048285	Organelle fission	24	2.76	4.77E-04	635	229	13,528	2.233	0.725015	0.094539	0.845916
GO:0007067	Mitosis	23	2.65	6.66E-04	635	220	13,528	2.227	0.834895	0.120723	1.178194
GO:0000280	Nuclear division	23	2.65	6.66E-04	635	220	13,528	2.227	0.834895	0.120723	1.178194
GO:0000087	M phase of mitotic cell cycle	23	2.65	8.47E-04	635	224	13,528	2.187	0.899066	0.141773	1.497678
GO:0000082	G1/S transition of mitotic cell cycle	10	1.15	0.001083	635	56	13,528	3.804	0.946655	0.167386	1.910126
GO:0051301	Cell division	27	3.11	0.001511	635	295	13,528	1.950	0.983250	0.203228	2.654958
GO:0000279	M phase	29	3.34	0.001709	635	329	13,528	1.878	0.990223	0.216170	2.999165

Pathways belonging to various cell functions were obtained from public external databases (http://www.geneontology.org/), and a Fisher’s exact test was performed to detect the significantly regulated pathways. Enrichment score: measure of pathway cluster enrichment over the other clusters; count: number of regulated genes within the pathway; *P*-value: significance of pathway enrichment; List Total: number of genes within the analyzed list of target genes having at least one GOBP annotation; Pop Hits: number of genes available on the entire microarray, annotated by the considered GOBP category or annotation cluster; Pop Total: number of genes available on the entire microarray having at least one GOBP annotation; Bonferroni: adjusted *P*-value by Bonferroni method; Benjamini: adjusted *P*-value by Benjamini method; FDR: false discovery rate.

Protein expression of Panc02 cells with stable shRNA expression prior to implantation into mice.

Chromatin immunoprecipitation from Capan-1 cells with or without siRNA-mediated knockdown of TBL1. IgG and primers for PIK3CA coding region served as a negative control; histone H3 served as a positive control.

Proliferation time course assay in control and TBL1 shRNA-transfected Panc02 cells overexpressing constitutively active PI3K p100α mutant E545K. Cells were stained with crystal violet, and absorbance was measured at 595 nm. *n *=* *9 cell culture wells per group; statistically significant at *P* < 0.05 for indicated comparisons: shNC pBabe-puro vs. shNC pBabe-PI3K (*), shTBL1 pBabe-puro vs. sbTBL1 pBabe-PI3K (#), shNC pBabe-puro vs. shTBL1 pBabe-puro ($), shNC pBabe-PI3K vs. shTBL1 pBabe-PI3K (†); *P*-values: day 2: 2.01 × 10^−3^ (#), 4.11 × 10^−9^ ($), 5.77 × 10^−3^ (†); day 3: 4.50 × 10^−5^ (*), 1.01 × 10^−9^ (#), 1.71 × 10^−30^ ($), 1.22 × 10^−6^ (†); day 4: 5.61 × 10^−22^ (*), 1.10 × 10^−45^ (#), 6.65 × 10^−62^ ($); two-way ANOVA with Bonferroni post-test.

Matrigel invasion assay in same cells as in (D). Invading cells per microscopy field were counted. *n *=* *6 microscopy fields per group; statistically significant at *P* < 0.05; *P*-values: 6.64 × 10^−4^ (*), 1.35 × 10^−7^ (#), 6.94 × 10^−5^ ($); two-way ANOVA with Bonferroni post-test.

mRNA expression of PIK3CA in human patient samples; significant difference (*P* < 0.05) between tumor and normal tissue *P = 2.13 × 10^−4^ determined by paired two-tailed Student’s *t*-test, *n *=* *18 patients.

Correlation of mRNA expression of TBL1 and PIK3CA in human patient tumor tissue (normalized to 18S rRNA and relative to a pooled organ donor sample); *n *=* *17 patients; *P*-value determined by testing for slope of regression line ≠ 0.

Survival analysis in 105 patients with PDAC: High PIK3CA expression is significantly associated with shorter post-resection survival. *P*-value determined by log-rank test as described in Materials and Methods. Data information: Data in (D) and (E) plotted as mean ± SEM.

Interestingly, the p110α catalytic subunit of phosphatidylinositide 3 (PI3) kinase appeared among the most strongly down-regulated genes upon TBL1 deficiency, the effect of which was conserved between human and mouse pancreatic cancer cells (Fig3B; Supplementary Fig S3C and E and S6A and B) and was specific for the p110α subunit as other PI3 kinase subunits remained unchanged (Supplementary Fig S6C). Indeed, an initial TBL1 chromatin immunoprecipitation coupled to massive parallel sequencing (ChIP-Seq) analysis in mouse liver identified a TBL1-binding site within the p110α PI3 kinase gene regulatory region (Supplementary Fig S8), and subsequent ChIP PCR studies using Capan-1 cells confirmed the recruitment of TBL1 to the p110α PI3 kinase gene promoter in pancreatic cancer cells (Fig3C). To functionally validate the importance of p110α PI3 kinase as a downstream mediator of TBL1 action, we performed genetic rescue experiments in murine Panc02 pancreatic cancer cells (Supplementary Fig S9). TBL1 inhibition substantially impaired cellular proliferation of these cells as shown above, while single p110α PI3 kinase expression had no major effect (Fig3D). Importantly, re-constitution of p110α PI3 kinase expression under conditions of TBL1 inhibition completely reversed the defect in cellular proliferation (Fig3D), demonstrating that p110α PI3 kinase indeed acts downstream of TBL1. Consistent with this notion, p110α PI3 kinase re-expression also rescued the impairment in invasive capacity of these tumor cells triggered by TBL1 knockdown (Fig3E).

In line with these data, p110α PI3 kinase was found to be significantly overexpressed in the original cohort of human PDAC patients (Fig3F), thereby significantly correlating with the mRNA expression levels of TBL1 in this cohort (Fig3G). Consistent with this correlation and the observed effect of TBL1 on survival (Fig1B), also PI3 kinase levels were found to be significantly associated with patient survival (Fig3H). Together with the *in vitro* data, these observations indicate that TBL1 represents a direct transcriptional regulator of p110α PI3 kinase and thus controls an essential node in the intracellular pro-tumorigenic network in PDAC (Eser *et al*, 2013) that may explain in some parts the multi-facetted impact of TBL1 on pancreatic cancer cell behavior.

### TBL1 inactivation prevents and reverses pancreatic tumor growth and increases chemosensitivity *in vivo*

Thus far, our data support the notion that TBL1 exerts a regulatory function upstream of the p110α PI3 kinase signaling network in pancreatic cancer cells, thereby controlling cell proliferation and metabolic adaptation.

**Figure 4:**
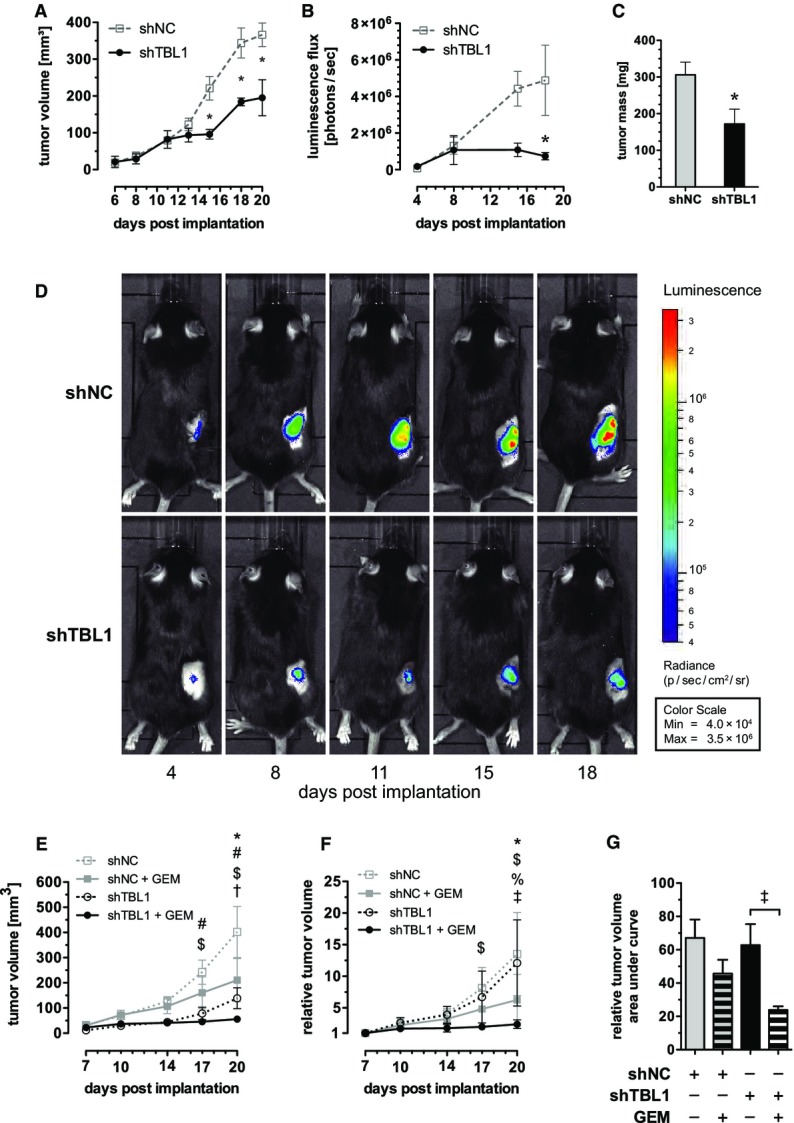
TBL1 reverses and prevents tumor growth *in vivo* and sensitizes toward chemotherapy A–D Panc02 cells with stable expression of luciferase were implanted subcutaneously into C57Bl6/N mice. Six days later, tumors were injected three times per week with 10^8^ ifu of adenovirus encoding shRNA. (A–C): Data plotted as mean ± SEM; *n *=* *4 animals in control (shNC) group and *n *=* *3 animals in knockdown (shTBL1) group; significantly different (*P* < 0.05) between control (shNC) and knockdown (shTBL1) *. (A) Tumor volume determined by transcutaneous measurement with digital calipers; *P*-values: 9.21 × 10^−3^ at day 15, 6.02 × 10^−4^ at day 18, 2.49 × 10^−4^ at day 20; two-way ANOVA with Bonferroni post-test. (B) Luminescence of tumors after intraperitoneal injection of firefly d-luciferin; *P* = 0.0133 at day 20; two-way ANOVA with Bonferroni post-test. (C) Tumor mass at necropsy; *P* = 0.0299, one-sided Welch’s *t*-test. (D) Luminescence images of one representative mouse in each group.

Consequently, we next aimed to address the consequences of TBL1 knockdown for tumor growth *in vivo*. To this end, we employed a heterotopic implantation mouse model injecting luciferase-positive murine Panc02 cells into the flank of wild-type C57BL/6 mice. An adenovirus containing TBL1-specific shRNA was injected directly into the tumors every 2–3 days starting from day 6 after tumor implantation, when tumors were palpable. Tumor growth was monitored over a 20-day period by *in vivo* luminescence imaging as well as transcutaneous measurement of tumor size. In congruence with *in vitro* data, tumor growth was substantially compromised in mice upon shRNA-mediated TBL1 knockdown in comparison to control shRNA-injected animals as evidenced by strongly diminished luminescence signal intensities and ultimate tumor size at the end of the experimental period (Fig4A–D; Supplementary Fig S10A).

E–G Panc02 cells with stable expression of shRNA were implanted subcutaneously into C57Bl6/N mice. Seven days later, mice were treated with 20 mg/kg gemcitabine delivered by intraperitoneal injection; *n *=* *4 animals in shNC+GEM group, *n *=* *5 animals in all other groups; significantly different (*P* < 0.05) between groups: shNC vs. shNC+GEM (*), shNC vs. shTBL1 (#), shNC vs. shTBL1+GEM ($), shNC+GEM vs. shTBL1 (%), shNC+GEM vs. shTBL+GEM (†); shTBL1 vs. shTBL1+GEM (‡); two-way ANOVA with Bonferroni post-test. (E) Tumor volume determined by transcutaneous measurement with digital calipers. *P*-values: day 17: 8.48 × 10^−3^ (#), 1.07 × 10^−3^ ($), day 20: 3.07 × 10^−3^ (*), 9.37 × 10^−6^ (#), 1.56 × 10^−8^ ($), 0.0239 (†). (F) Tumor volume relative to the volume at day 7 post-implantation; *P*-values: day 17: 3.94 × 10^−3^ ($), day 20: 1.09 × 10^−3^ (*), 1.39 × 10^−7^ ($), 0.01195 (%), 3.12 × 10^−6^ (‡). (G) Area under curve for relative tumor volume; *P* = 0.0195 (‡). Data information: All data plotted as mean ± SEM.

**Figure 5:**
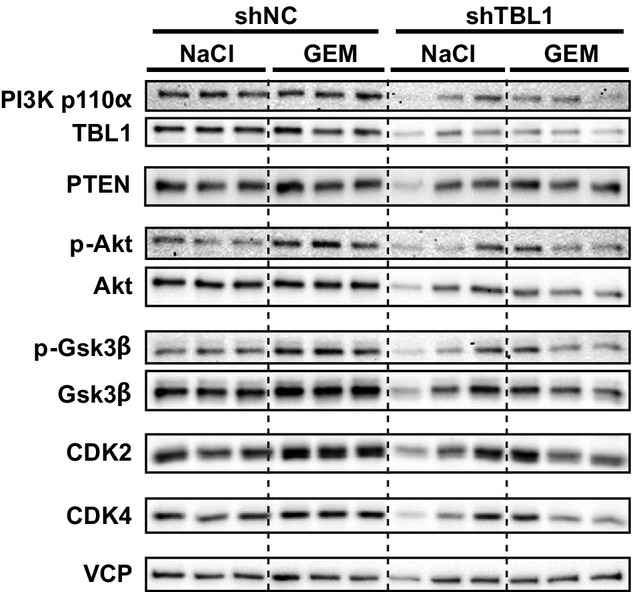
TBL1-depletion leads to reduction in PI3 kinase p110α and cell cycle-associated proteins Panc02 cells with stable expression of shRNA were implanted subcutaneously into C57Bl6/N mice. Seven days later, mice were treated with 20 mg/kg gemcitabine delivered by intraperitoneal injection. After 21 days, mice were sacrificed, tumors were removed and proteins were extracted and immunoblotted.

To complement the above-described “therapeutic” approach by a preventive experimental setup, we established Panc02 cell lines stably expressing TBL1-specific or non-specific control shRNA by lentiviral gene delivery (Fig3B). In addition, utilization of glucose as a primary energy source of tumor cells has recently been reported to promote chemoresistance of tumors, thereby limiting prospects of cancer therapy. In turn, glucose deprivation was found to enhance toxicity of chemotherapeutics and ionizing radiation for cancer cells (Simons *et al*, 2007; Aghaee *et al*, 2012). Since our data thus far indicated that TBL1 ablation may prevent glucose consumption in pancreatic cancer cells (Fig2C and D), we combined the “preventive” setup with the question whether TBL1 deficiency may also enhance chemosensitivity of pancreatic tumor cells. After transplantation of identical cell numbers of both control and TBL1-depleted cells into the flanks of wild-type C57BL/6 mice, distinct animal cohorts were treated with gemcitabine as the most commonly used chemotherapeutic drug in PDAC, and tumor size was monitored for the duration of 3 weeks. In line with a chemosensitizing impact of TBL1, sensitivity of TBL1-deficient tumors to gemcitabine was significantly enhanced as indicated by reduced tumor size when compared with shNC-treated cells (Fig4E–G; Supplementary Figs S10B and S11). Consistent with a direct transcriptional regulation of p110α PI3 kinase expression, TBL1 knockdown was able to also impair p110α PI3 kinase protein levels under these conditions as well as the activity of prototypical PI3 kinase targets, including protein kinase B/Akt and glycogen synthase kinase (GSK) 3, in pancreatic cancer cells, while levels of the PI3 kinase antagonist PTEN remained unchanged (Fig5; Supplementary Fig S12). These data demonstrated that tumor-specific inactivation of TBL1 sensitized pancreatic cancer to chemotherapeutic agents and diminished tumor growth *in vivo* in both therapeutic and preventive settings using two independent technical approaches. These studies overall suggested that high TBL1 expression levels in pancreatic tumors promote disease progression *in vivo*. TBL1 may thus serve as a prognostic biomarker for aggressiveness and/or therapy responsiveness in human PDAC and may represent a novel molecular target for future therapeutic approaches.

## Discussion

Our study identifies TBL1 as an integral regulator of PDAC biology, controlling tumor cell proliferation, invasion, and PI3 kinase signaling of pancreatic cancer cells.

As a component of the tumorigenic PDAC program in both mice and humans, TBL1 overexpression may contribute to poor therapy responsiveness and high malignancy of human PDAC. Thereby, our data are in line with an earlier unbiased transcriptome analysis reporting that TBL1 is up-regulated in certain populations of micro-dissected tumors (Jones *et al*, 2008), indicating that the induction of TBL1 may represent a robust feature of malignant pancreatic cells in distinct patient cohorts. Previous studies in other tissues, most notably liver and white adipose tissue, have shown that TBL1 expression levels are controlled through fatty acid and cAMP-dependent pathways, respectively (Kulozik *et al*, 2011; Rohm *et al*, 2013). Interestingly, preliminary data point toward the possibility that also in pancreatic tumor cells, TBL1 may act downstream of hormonal signal transduction, including cAMP and insulin (Stoy, Strobel and Herzig, unpublished). It will be interesting to determine whether and how these endocrine cues interact with the mutant KRAS pathway to induce TBL1 gene transcription in the PDAC setting.

TBL1 was originally cloned in relationship to an X-linked human disorder, Ocular Albinism with late-onset Sensorineural Deafness (OASD), in which deletion of TBL1 was suggested to be responsible for the hearing defect (Bassi *et al*, 1999). In metabolism, we have identified the TBL1/TBLR1 transcriptional co-factor complex as a key regulator of hepatic lipid homeostasis and fatty liver disease with dysfunctional activities in mono- and multigenic diabetes, in both murine as well as human setting (Kulozik *et al*, 2011). In addition, our subsequent studies demonstrated that the genetic inactivation of TBLR1 blunts the lipolytic response of white adipocytes through the impairment of cAMP-dependent signal transduction. Indeed, mice lacking TBLR1 in adipocytes are defective in fasting-induced lipid mobilization and when placed on a high-fat diet show aggravated adiposity, glucose intolerance, and insulin resistance (Rohm *et al*, 2013). The combined data in both liver and adipose tissue underlined the notion that this specific transcriptional co-factor complex may represent a critical determinant of systemic lipid handling by coordinating energy substrate usage in different tissue locations.

In this respect, alterations in cellular metabolism have “re-emerged” as a hallmark of cancer (Hanahan & Weinberg, 2011), most notably including the so-called Warburg effect or aerobic glycolysis (Warburg, 1956). More specifically, tumor cells, since they are highly proliferative, switch to glycolysis even under aerobic conditions in order to meet their increased demand for nucleotide and amino acid building blocks. Consequently, numerous tumor tissues are characterized by a dramatically increased uptake of glucose and/or glutamine (Ward & Thompson, 2012). Interestingly, glucose deprivation has been demonstrated to also enhance chemosensitivity of certain tumors, most probably via interference with the availability of sufficient energy substrates for effective DNA damage responses (Simons *et al*, 2007; Aghaee *et al*, 2012; Dittmann *et al*, 2013).

Our current study now adds a new layer to TBL1-dependent metabolic control as it points toward a potential role of TBL1 in metabolic regulation of pancreatic cancer cells, that is, enhancement of cellular glucose uptake and aerobic glycolysis, concomitant with a potential role in defining the chemosensitivity status. While these metabolic alterations could be secondary consequences of TBL1 effects on proliferative pathways and deserve further attention in future studies, it is still tempting to speculate that the TBL1 transcriptional co-factor complex defines a conserved “metabolic” checkpoint between non-malignant and cancer cells, controlling essential nodes in metabolic pathways under both homeostatic (fasting-feeding cycles) and malignant conditions.

Of note, TBL1 has also been linked to the oncogenic Wnt signaling pathway in other tumor types by the observation that the recruitment of TBL1 and β-catenin to Wnt target gene promoters is mutually dependent (Li & Wang, 2008). TBL1 might therefore act as a key integrating component of the PDAC tumorigenic program by determining both glucose usage and PI3 kinase-mediated proliferative and metabolic responses. Indeed, tumor-specific TBL1 inactivation was sufficient to either reverse disease burden of established tumors or even prevent tumor development at an earlier stage, correlating with enhanced tumor susceptibility to chemotherapeutic intervention. Noteworthy, the absence of changes in Wnt target gene expression upon TBL1 deficiency (Supplementary Table S2) suggested that this previously described TBL1 target pathway may be of minor relevance for TBL1-dependent tumorigenesis in PDAC as compared with other tumor entities and the significant regulatory function of TBL1 for the PI3 kinase signaling pathway.

Taken together, targeted modulation of TBL1 transcriptional co-factor activity supportive to chemotherapy may thus represent a novel option for improved PDAC treatment. This assumption is supported by our human data showing an association of PDAC TBL1 levels and survival, as well as a correlation to PI3 kinase expression, respectively. Chemoresistance and genetic heterogeneity of PDAC also demand new approaches toward patient stratification in order to develop tailored therapy options. Our results now indicate that TBL1 expression levels may serve as a novel potential marker and molecular target for PDAC therapy in the future.

## Materials and Methods

### Materials

All chemicals were purchased from Sigma-Aldrich unless stated otherwise. Cell culture media and supplements were from Gibco Life Technologies unless stated otherwise. Cell culture plastic ware was obtained from BD unless stated otherwise.

### Animal experiments

p48-Cre; LSL-Kras^G12D^ mice were bred in the C57BL/6N background and genotyped as previously described (Hingorani *et al*, 2003). All mice were maintained on a 12-h light-dark cycle at 24°C with regular unrestricted diet. Wild-type male C57BL/6N mice were injected with 2 × 10^5^ luciferase-expressing or shRNA-expressing Panc02 murine pancreatic cancer cells in 100 μl DPBS. Body weight was monitored every 2–3 days. Tumors were measured transcutaneously every 2–3 days with digital calipers, and volume was calculated with the formula for ellipsoids (*V* = 4/3 π × (width/2)^2^ × (length/2)^2^). For luminescence-based measurement of tumor growth, animals were injected intraperitoneally with 10 μl/g body weight of 15 mg/ml firefly d-luciferin in DPBS and anaesthetized with 3% isoflurane/O_2_. Ten minutes after luciferin injection, animals were placed in an IVIS Lumina II (Caliper Life Sciences) under 1.5% isoflurane/O_2_ and luminescence was measured for 5 min. Adenovirus was produced as previously described (Herzig *et al*, 2001), and 10^8^ ifu in 20 μl DBPS/10% glycerol were injected directly into tumors every 2–3 days using a microinjector (Narishige, London, UK). shRNA sense strand sequences are as follows: shNC GATCTGATCGACACTGTAATG, shTBL GCGAGGATATGGAACCTTAAT.

In each animal experiment, mice were randomly assigned to each group. Number of animals per group to detect biologically significant effect sizes was calculated using appropriate statistical sample size formula and indicated in the biometrical planning section of the animal license submitted to the governing authority. Blinding was not done during animal group allocation. In subcutaneous implantations, animals were excluded from further analysis if tumors had invaded the peritoneal cavity. Animal handling and experimentation was performed in accordance with the European Union directives and the German animal welfare act (Tierschutzgesetz) and approved by local authorities (Regierungspräsidium Karlsruhe, Germany).

### Lentivirus transduction

HEK293T cells were transfected with 1 μg of lentiviral pLKO.1 vector with TBL1-specific shRNA (SHCLNG-NM_020601, Sigma) or scrambled shRNA (Addgene, #1864), 1 μg psPAX2 (Addgene, #12260) and 100 ng pMD2.G (Addgene, #12259) in a 6-well format using Lipofectamine 2000 according to manufacturer’s instructions. The following day, medium was renewed and supplemented with 1.1% BSA. Supernatant was harvested 48 h later by centrifugation, and titer was determined with HIV p24 ELISA (XpressBio, Thurmont, MD, USA). Panc02 cells were infected with viral supernatant supplemented with 8 μg/ml polybrene at an MOI of 10, and a stable population was selected with 2 μg/ml puromycin. Cells were grown to sufficient amounts and injected subcutaneously into C57BL/6N mice as described.

### Retroviral infection

Ecotropic retroviruses were generated by overnight co-transfection of HEK293T cells with 4 μg pBabe-puro (Addgene 1764) or pBabe-PI3KE545K (Addgene 12525) together with 2 μg of pUMVC (Addgene 8448) and 1 μg of pMD2.G (Addgene 12259) vectors. PancO2 shScrambled and shTBL1 cells were infected by incubating with HEK293T cell supernatant containing retrovirus supplemented with 4 μg/ml polybrene. Cells were allowed to grow for 48 h post-infection and were seeded for growth curve or processed for protein extraction and Western blot.

### Histology

Tissues were collected and immediately stored in 4% phosphate-buffered formaldehyde for 18–24 h. Tissues were embedded in paraffin using standard procedures. Four-micrometer sections were stained with hematoxylin and eosin following standard procedures or with specific antibodies for TBL1 and Ki-67 and counterstained with hematoxylin.

### Cell culture

All cell lines were grown in high-glucose (4.5 g/l) DMEM (Gibco, Darmstadt, Germany) supplemented with 10% fetal bovine serum (Gibco) and 1% penicillin–streptomycin (Gibco) in a humidified incubator at 37°C and 5% CO_2_. Capan-1 cells were obtained from ATCC. Panc02 cells with stable expression of luciferase were kindly provided by Dr. Ana Martin-Villalba (DKFZ). Panc8680 cells were kindly provided by Jens Siveke (TU, Munich). The absence of contamination was confirmed by Multiplexion (Heidelberg, Germany) using the Multiplex Cell contamination Test (McCT) (Schmitt & Pawlita, 2009).

### siRNA transfection

Cells were plated and transfected 24 h later with 0.5 pmol (Capan-1) or 2 pmol (Panc8680) siRNA per 1,000 cells using Lipofectamine 2000 (Invitrogen) following manufacturer’s instructions (human TBL1: SI04296 (target sequence TGGCGCGTAGTAAGTGCTTTA, referred to as siTBL1 #1) and SI04329514 (target sequence TGCGTTAGAGTGTACTCTGAA referred to as siTBL1 #2), mouse TBL1: SI01443036 (target sequence CAGGAGCCTGTCTATAGTGTA), unspecific siRNA: 1027310 (referred to as siNC #1), and 1027292 (referred to as siNC #2), all from Qiagen). Culture media was renewed 24 h after application of siRNA.

### 2-Deoxy-glucose uptake

Capan-1 cells were transfected with siRNA against TBL1 as described above. Twenty-four hours after transfection, medium was replaced, and another 24 h later, media was changed to pre-gassed and pre-warmed Krebs-Ringer-Henseleit (KRH) buffer containing 118.5 mM NaCl, 24.65 mM NaHCO_3_, 4.74 mM KCl, 1.18 mM MgSO_4_, 1.18 mM KH_2_PO_4_, 2.5 mM CaCl_2_, 1 mM Hepes (pH 7.4), 25 mM glucose, 0.1% BSA, 1× RPMI amino acids, 1 mM pyruvate, 2 mM glutamine, and 8 mM mannitol, and cells were pre-incubated for a further 2 h before incubating with tracer media (pre-gassed and pre-warmed KRH buffer containing 118.5 mM NaCl, 24.65 mM NaHCO_3_, 4.74 mM KCl, 1.18 mM MgSO_4_, 1.18 mM KH_2_PO_4_, 2.5 mM CaCl_2_, 1 mM Hepes (pH 7.4), 0.1% BSA, 1× RPMI amino acids, 1 mM pyruvate, 2 mM glutamine, 8 mM mannitol + ^14^C-mannitol (NEC314050UC, Perkin Elmer; specific activity ≈250 dpm/nmol), 1 mM 2-deoxyglucose + ^3^H-2-deoxyglucose (NET549001MC, Perkin Elmer; specific activity ≈500 dpm/nmol) for exactly 15 min.

Tracing was stopped by removing tracer media and immediately adding ice-cold pre-incubation media. Cells were lysed in 300 μl RIPA buffer with protease inhibitor. 200 μl lysate as well as tracer media (to calculate the specific activity of ^3^H-2-deoxyglucose and ^14^C-mannitol) and lysis buffer (background radiation) were counted by scintillation counting (^3^H and ^14^C dual DPM). A total of 10 μl of lysate was used in duplicate to quantify protein content for normalization. 2-deoxyglucose uptake rates were calculated based upon incorporation of ^3^H counts into cells versus the specific activity of 2-deoxyglucose in the media, with correction for extracellular space by mannitol tracing.

### Cell growth assays

Cell growth was assessed with the Click-iT® EdU Imaging Kit Alexa-Fluor 555 (Invitrogen) according to manufacturer’s instructions. Alternatively, cell number was determined with crystal violet. To assess relative cell numbers, cells were grown in 24-well plates (10,000 cells/well). At 1, 2, 3, and 4 days, cells were washed with PBS, fixed with methanol: acetic acid (3:1) for 20 min, stained with crystal violet for an additional 20 min, washed five times with PBS, and air-dried. Dye was eluted from cells at room temperature by the addition of 1 ml of 10% acetic acid, and absorbance was measured at 595 nm.

### Matrigel Invasion assay

A total of 40,000 Panc02 cells with stable shRNA expression or 100,000 siRNA-transfected Capan-1 cells were seeded in 100 μl serum-free DMEM with 1% penicillin–streptomycin on the top chamber of a matrigel-coated transwell invasion chamber (#354480, Corning), and 600 μl of DMEM with 10% FBS and 1% penicillin–streptomycin was added to the bottom chamber. Serum-free DMEM with 1% penicillin–streptomycin was used as a negative control. Cells were incubated at 37°C and 5% CO_2_ for 16 h (Panc02) or 24 h (Capan-1). Non-invading cells were removed, and invading cells were fixed with methanol for 5 min and stained with 0.1% crystal violet for 15 min before imaging and quantification.

### FACS analysis

Of 2.5 × 10^5^ cells were seeded in 6-well plates, transfected as described, and collected by trypsinization 48 h later. Cells were washed once with PBS, resuspended in 200 μl cold PBS, and added dropwise to 4 ml of cold 70% ethanol while vortexing. Cells were fixed in ethanol for 2–24 h at 4°C, then centrifuged, resuspended in staining buffer (40 μg/ml propidium iodide, 100 μg/ml RNase A, 0.1% Triton X-100, in PBS), and incubated for 30 min at 37°C. Finally, cells were strained and measured in a FACSORT (BD, Heidelberg, Germany). Using Weasel software (version 3.0), single cells were gated and cell cycle phases were quantified with the software’s cell cycle curve fitting functionality.

### Extracellular flux analysis

Cells were seeded at 1 × 10^4^ per well in 96-well polystyrol Seahorse Plates. siRNA-mediated knockdown was performed the following day as described. Mito Stress Test Kit or Glycolysis Stress Test Kit was used following manufacturer’s instructions (Seahorse Bioscience, Copenhagen, Denmark) with the following pre-tested assay chemical concentrations: 2 μM oligomycin, 0.5 μM FCCP, 1 μM rotenone, 1 μM antimycin A, 10 mM glucose, and 100 mM 2-deoxyglucose. After completion of measurement, medium was aspirated and cells were fixed in ice-cold 5% acetic acid/95% ethanol for at least 30 min. Then plate was washed twice with water, and cells were stained with 50 μl per well of 0.4% sulforhodamine B (SRB) in 5% acetic acid for 30 min protected from light. Unbound SRB was removed by washing four times with 1% acetic acid, and then SRB bound to cells was eluted with 100 μl per well of 10 mM unbuffered Tris for 10 min. Absorbance was read at 550 nm and converted to cell number using a standard curve. Measurement data were normalized relative to cell number.

### Chromatin immunoprecipitation assay

Cells were fixed for 15 min with 1% formaldehyde in culture medium on 15 cm culture plates, fixation was stopped with 125 mM glycine for 5 min, and medium was removed and washed twice with cold DPBS. Cells were scraped off in 2 ml 0.5 M PMSF in DPBS and resuspended, centrifuged for 5 min at 1,500 rpm and 4°C. The pellet was resuspended in 5 ml cold ChIP swelling buffer (25 mM HEPES pH 7.2; 1 mM MgCl_2_; 10 mM KCl; 1% IGEPAL; 1 mM DTT; 0.5 mM PMSF; 1× protease inhibitor cOmplete (Roche)), incubated on ice for 10 min, homogenized in a Dounce tissue grinder, incubated for 10 min at 4°C, and centrifuged at 2,000 rpm for 5 min at 4°C. The pellet was resuspended in 5 ml of ChIP Sucrose buffer A (320 mM sucrose; 15 mM HEPES, pH 7.9; 60 mM KCl; 2 mM EDTA; 0.5 mM EGTA; 0.5% BSA; 0.5 mM spermidine; 0.15 mM spermine; 0.5 mM DTT; final pH 7.2) and homogenized in a Dounce tissue grinder. The resulting nuclear suspension was carefully layered over 5 ml of ChIP Sucrose buffer B (416 mM sucrose; 15 mM HEPES, pH 7.9; 60 mM KCl; 2 mM EDTA; 0.5 mM EGTA; 0.5% BSA; 0.5 mM spermidine; 0.15 mM spermine; 0.5 mM DTT; final pH 7.2) and centrifuged at 3,000 rpm for 15 min at 4°C. Pellet was resuspended in 1 ml of MNase digestion buffer (50 mM Tris, pH 7.4; 25 mM KCl; 4 mM MgCl_2_; 1 mM CaCl_2_; 1× EDTA-free protease inhibitor cOmplete (Roche)) and centrifuged at 3,000 rpm for 5 min at 4°C, then resuspended in MNase digestion buffer to an OD_260_ of 0.2. For chromatin fragmentation, 8 μl of micrococcal nuclease (NEB) were added and incubated for 20 min at 37°C, and mixed by inverting every 3–5 min. The reaction was stopped by adding EDTA to a final concentration of 10 mM and putting on ice. Nuclei were pelleted by centrifuging at 13,000 rpm for 1 min at 4°C, resuspended in 1 ml of ChIP buffer (50 mM Tris, pH 8.0; 140 mM NaCl; 1 mM EDTA; 1% Triton X-100; 0.1 mM sodium deoxycholate; 0.1% SDS; 0.5 mM PMSF; 1× protease inhibitor cOmplete (Roche)) and incubated on ice for 10 min. Sample was centrifuged at 13,000 rpm for 15 min at 4°C, and the chromatin-containing supernatant was transferred to a fresh tube. Concentration of fragmented cross-linked chromatin was measured with a Qubit® 2.0 Fluorometer (Invitrogen) using the Qubit® dsDNA HS Assay Kit (Invitrogen) following manufacturer’s instructions. A total of 10 μg of chromatin was used for precipitation in a final volume of 500 μl ChIP buffer plus protease inhibitors. Lysates were pre-cleared with 30 μl of ChIP-grade protein G agarose beads (#9007S, Cell Signaling) and 4 μg Normal Rabbit IgG (#2729, Cell Signaling) for at least 2 h on a rotating wheel at 4°C. Tubes were centrifuged at 6,000 rpm for 1 min at 4°C, and supernatant was transferred to a new tube. Antibodies were added as follows and incubated over night at 4°C: 1 μg negative control Normal Rabbit IgG (#2729, Cell Signaling), 10 μl positive control Histone H3 antibody (#4620, Cell Signaling), and 10 μl TBL1X-antibody (ab24548, Abcam). A total of 30 μl ChIP-grade protein G agarose beads were added and incubated for 2 h on a rotating wheel at 4°C, and then centrifuged at 6,000 rpm for 1 min at 4°C. Supernatant was aspirated, and the beads were washed twice with 1 ml of ChIP wash buffer A (50 mM Tris; 140 mM NaCl; 1 mM EDTA; 1% Triton X-100; 0.1% sodium deoxycholate; 0.1% SDS), twice with 1 ml of ChIP wash buffer B (50 mM Tris; 500 mM NaCl; 1 mM EDTA; 1% Triton X-100; 0.1% sodium deoxycholate; 0.1% SDS), twice with 1 ml of ChIP wash buffer C (20 mM Tris; 250 mM LiCl; 1 mM EDTA; 0.5% IGEPAL; 0.1% sodium deoxycholate) and three times with 1 ml 1× TE with a 5-min rotation at room temperature for each washing step. DNA was eluted twice by adding 100 μl ChIP elution buffer (50 mM Tris, pH 8.0; 1 mM EDTA; 0.1% SDS; 50 mM NaHCO_3_), incubating at 65°C and 1,200 rpm on a shaking heating block for 15 min followed by centrifugation at 13,000 rpm for 1 min at room temperature. Both supernatants were pooled, and 8 μl of 5 M NaCl was added and incubated at 65°C over night at 950 rpm on a shaking heating block. Proteinase K was added to a final concentration of 1 mg/ml and incubated at 56°C for 2 h at 950 rpm on a shaking heating block. RNase A was added to a final concentration of 100 μg/ml and incubated at 37°C for 1 h followed by inactivation at 95°C for 10 min. DNA was extracted with the MinElute PCR purification kit (Qiagen) following manufacturer’s instructions.

Precipitated DNA fragments were analyzed by SYBR Green quantitative PCR amplification using primers directed against the PIK3CA promoter region (forward: GGCGGAAAAGCAAGACGCA; reverse: AAGAAGCGGAAGCGAAATTGAGGC) or coding region (forward: GACTTAGGCAAGGCTGTAGAGTA; reverse: GTGTTCAGTGTACAGGTTTGTGTAT).

### Protein analysis

Frozen organ samples were homogenized with stainless steel beads in ice-cold lysis buffer (50 mM Tris pH 7.2, 1 mM EDTA, 10 mM NaF, 2 mM Na_3_VO_4_, 1 mM DTT freshly supplemented with 1× protease inhibitor (P8340, Sigma-Aldrich) and 1× phosphatase inhibitor (P5726, Sigma-Aldrich)) using a TissueLyser (Qiagen) at 30 Hz for 1–3 min, then supplemented with 150 mM NaCl and 1% NP-40, and mixed on a rotating wheel at 4°C for 1 h. Cellular debris was removed by centrifuging for 10 min at 13,000 rpm in a tabletop centrifuge. Cultured cells were washed with PBS, lysed with ice-cold RIPA buffer (50 mM Tris, 250 mM NaCl, 2% NP-40, 2.5 μM EDTA, 0.1% SDS, 0.5% sodium deoxycholate freshly supplemented with 1× protease inhibitor (P8340, Sigma-Aldrich) and 1× phosphatase inhibitor (P5726, Sigma-Aldrich)), sonicated at low intensity with two cycles of 30 s ON / 30 s OFF in a Bioruptor Plus (Diagenode), and cellular debris was removed by centrifuging for 10 min at 13,000 rpm in a tabletop centrifuge. Protein concentration was determined with the BCA kit from Thermo Scientific. Extracts were separated on 10% SDS–polyacrylamide gels and blotted onto nitrocellulose membranes. Western blot assays were performed using antibodies specific for TBL (ab24548, Abcam), PI3 kinase p100α (#4249, Cell Signaling), PTEN (#9552, Cell Signaling), phospho-PTEN (#9549, Cell Signaling), Akt (#9272, Cell Signaling), phospho-Akt(#9271, Cell Signaling), GSK3β (#9315, Cell Signaling), phospho-GSK3β (#9336, Cell Signaling), CDK2 (sc-163, Santa Cruz), CDK4 (#2906, Cell Signaling), VCP (ab11433, Abcam), or β-actin (A5441, Sigma-Aldrich).

### Quantitative Taqman RT-PCR

Total RNA was extracted from frozen tissue samples of mice or the initial patient cohort tissue samples (Figs1A and 3F and G) or cultured cells using the RNeasy (Qiagen, Hilden) kit. cDNA was prepared by reverse transcription using M-MuLV enzyme and Random Hexamer primers (Fermentas, St. Leon-Rot). cDNAs were amplified using assay-on-demand kits and an ABI StepOnePlus sequence detector (Applied Biosystems, Darmstadt). RNA expression data were quantified according to the delta C_T_ method as described (Livak & Schmittgen, 2001) and normalized to levels of TATA-box-binding protein RNA (TBP) for mouse tissue and cultured cells and to 18S-rRNA for human patient samples.

### Affymetrix mRNA microarray of siRNA-transfected Capan-1 cells

Total RNA was extracted as described above. cDNA synthesis was performed using the SuperScript Choice System (Invitrogen Life Technologies, Invitrogen Corporation) according to manufacturer’s protocol. Biotin-labeled cRNA was produced using ENZO BioArray HighYield RNA Transcript Labeling Kit. Standard protocol from Affymetrix (Santa Clara, CA) with 3.3 μl of cDNA was used for the *in vitro* transcription (IVT). Cleanup of the IVT product was done using CHROMA SPIN-100 columns (Clontech, USA). Spectrophotometric analysis was used for quantification of cRNA with acceptable A260/A280 ratio of 1.9 to 2.1. After that the cRNA was fragmented using Affymetrix defined protocol. Labeled and fragmented cRNA was hybridized to Affymetrix HG-U133 Plus 2.0 microarrays for 16 h at 45°C using Affymetrix defined protocol. Microarrays were washed using an Affymetrix fluidics station 450 and stained initially with streptavidin–phycoerytherin. For each sample, the signal was further enhanced by incubation with biotinylated goat anti-streptavidin followed by a second incubation with streptavidin–phycoerytherin and a second round of intensities were measured. Microarrays were scanned with Affymetrix scanner controlled by Affymetrix Microarray Suite software. A Custom CDF version 14 with Entrez based gene definitions was used to annotate the arrays. The raw fluorescence intensity values were normalized applying quantile normalization. Differential gene expression was analyzed based on log-linear mixed model ANOVA (Hsieh *et al*, 2003; Roy, 2007), using a commercial software package SAS JMP7 Genomics, version 5, from SAS (SAS Institute, Cary, NC, USA). A false-positive rate of a =0.05 with FDR correction was taken as the level of significance.

The over-representation analysis (ORA) is a microarray data analysis that uses predefined gene sets to identify a significant over-representation of genes in data sets (Subramanian *et al*, 2005; Manoli *et al*, 2006) Pathways belonging to various cell functions such as cell cycle or apoptosis were obtained from public external databases (KEGG, http://www.genome.jp/kegg/). A Fisher’s exact test was performed to detect the significantly regulated pathways.

Gene Set Enrichment Analysis (GSEA) was used to determine whether defined lists (or sets) of genes exhibit a statistically significant bias in their distribution within a ranked gene list (see http://www.broadinstitute.org/gsea/ for details (Subramanian *et al*, 2005). Pathways belonging to various cell functions such as cell cycle or apoptosis were obtained from public external databases (KEGG, http://www.genome.jp/kegg/).

The raw and normalized data are deposited in the Gene Expression Omnibus database (http://www.ncbi.nlm.nih.gov/geo/; accession No. GSE59761).

Analyses were done in the R version 2.15 environments. The ICC calculation was done using the irr-package.

### Human subjects

Human samples and clinical data are derived from a prospective tissue database of the European Pancreas Center (EPC), Department of General Surgery, University Hospital Heidelberg and were collected with informed consent as approved by the local ethics committee.

For the initial analysis, biopsies derived from resected specimen were analyzed from pathologically confirmed pancreatic ductal adenocarcinoma (tumor), adjacent tumor-free pancreas tissue (non-tumor), and chronic pancreatitis. Biopsies from pancreases harvested for organ donation but not allocated for transplantation served as a further control group (normal). Tissue was processed and analyzed as described above.

The survival analyses are based on biopsies from resected specimen from a larger collective of 105 patients with pancreatic ductal adenocarcinoma.

Frozen tissue samples from the 105 patient cohort were cut into slices of 10–20 μm thicknesses with a cryotome Leica CM 1850 UV at −34°C. The slices were submerged in liquid nitrogen and gently ground by three turns with a polypropylene micropestle (Eppendorf, Hamburg, Germany) that fits into 2 ml Eppendorf tubes. Total RNA was isolated with the AllPrep Isolation kit (Qiagen, Hilden, Germany), following the protocol suggested by the manufacturer. RNA integrity was evaluated using an Agilent 2100 Bioanalyzer (Agilent Technologies, Palo Alto, USA). Only RNA with a RNA Integrity Number of at least seven was used for microarray analysis. Total RNA from individual samples was analyzed on the Sentrix Human-6v3 Whole Genome Expression BeadChips (Sentrix Human WG-6; Illumina). To synthesize first and second-strand cDNA and for amplifying biotinylated cRNA, the Illumina Totalprep RNA Amplification Kit was used. Hybridization to the BeadChip was performed according to the manufacturer’s instructions without modification. In short, a maximum of 10 μl cRNA was mixed with a 20 μl GEX-HYB hybridization solution. The preheated 30 μl assay sample was dispensed onto the large sample port of each array and incubated for 18 h at 58°C. Subsequently, the samples were washed and scanned with a BeadArray Reader (Illumina, San Diego, CA). Raw data were exported from the Illumina Beadstudio software to R (Ritchie *et al*, 2011). The data were quantile-normalized and log_2_-transformed. To adjust for effects of variable acinar cell content in pancreatic biopsies, expression data were normalized using expression levels of the acinar cell-specific marker Pancreatic Lipase-Related Protein 2 (PNLIPRP2) (Heller *et al*, 2010). On logarithmized expression data, a correction factor was calculated by subtracting the expression of PNLIPRP2 in a given sample from median PNLIPRP2 expression. To normalize for PNLIPRP2 expression, this correction factor was added to quantile-normalized and log_2_-transformed TBL1 and PIK3CA expression data.

### Statistical analysis

Statistical analyses were performed with GraphPad Prism (version 5.04 for Windows, GraphPad Software, San Diego, CA, USA) using one-way or two-way ANOVA, Student’s *t*-test, Welch’s *t*-test, or linear regression analysis where appropriate. *P* ≤ 0.05 was considered statistically significant. Error bars in figures represent standard error of the mean (SEM) unless stated otherwise.

For the survival analyses, SAS (release 9.5) was used and the Kaplan–Meier method was applied. Patients alive at the last follow-up were censored. The median survival times are presented. Cutoff values for TBL1 and PIK3CA expression were determined by logistic regression analysis. The log-rank test was used to compare survival curves of subgroups. Two-sided *P*-values were computed and a difference was considered statistically significant at *P* ≤ 0.05.

**The paper explained**

**Problem**

Malignancy of the exocrine pancreas, in particular ductal adenocarcinoma (PDAC), is a detrimental disease with high lethality and limited therapeutic options. This severe form of cancer causes more than 200,000 deaths worldwide every year and has a 5-year survival rate of only 4%. It is therewith the fourth leading cause of cancer fatalities in Western societies. Due to the lack of prognostic markers, its remarkable resistance to chemotherapy, and the severity of the disease, the identification of novel potential targets in PDAC remains a main challenge in oncological research.

**Results**

Our study shows that transcriptional co-factor Transducin beta-like (TBL) 1 was overexpressed in both human and murine PDAC. Inactivation of TBL1 in human and mouse pancreatic cancer cells reduced cellular growth and aggressiveness and also regulated the activity of the oncogenic PI3 kinase signaling pathway *in vitro* and *in vivo*. Finally, TBL1 mRNA levels were also found to correlate with PI3 kinase levels and overall survival in a cohort of human PDAC patients.

**Impact**

Our study for the first time identifies TBL1 as an oncogenic driver in PDAC, thereby now expanding TBL1’s known metabolic functions in liver and adipose tissue to malignant conditions. Also, given the correlation of TBL1 levels with patient survival in a human PDAC cohort, our results suggest that TBL1 could be used as a prognostic or diagnostic marker for PDAC in the future. Finally, this study is likely to be of relevance also for other, extra-pancreatic malignancies as PI3 kinase signaling has been found to be altered in numerous tumor entities.

## Supporting Information

**Supplementary Table S2**

**Review Process File**
