# Supplementary material for: Transcriptional co-factor Transducin beta-like (TBL) 1 acts as a checkpoint in pancreatic cancer malignancy
Source: EMBO Mol Med. 2015 Jun 13;7(8):1048–62. doi: 10.15252/emmm.201404837 (PMC4551343; doi:10.15252/emmm.201404837)
Supplement: Supplementary file 1 [file emmm0007-1048-sd1.pdf]

SUPPLEMENTARY INFORMATION

Table of Contents

Supplementary Figure Legends S1-S12

Supplementary Figures S1-S12

SUPPLEMENTARY LEGENDS

SUPPLEMENTARY FIGURE LEGENDS

Fig. S1. Immunohistochemistry negative control

Negative control of immunohistochemistry staining without primary antibody in human (A) or mouse (B) pancreas.

Fig. S2. TBL1 protein is up-regulated in p48$^{+/Cre}$; Kras$^{+/LSL-G12D}$ mice

Immunoblot of pancreas tissue from p48$^{+/Cre}$; Kras$^{+/LSL-G12D}$ and wild type (wt) mice

Fig. S3. TBL1 siRNA validation

Expression of TBL1-mRNA after siRNA-mediated knockdown in human Capan-1 (A) and murine Panc8680 (B) cells. n=2 cell culture wells per group; significantly different ($p < 0.05$) from control (no siRNA) *, (siNC #1) #, (siNC #2) $; p-values panel (A): siNC #1: 24h 2.73×10^{-3} (*), 48h 2.31×10^{-3} (*), 72h 6.01×10^{-6} (*), siNC #2: 24h 4.33×10^{-7} (*), 48h 2.31×10^{-3} (*), 72h 1.49×10^{-9} (*), siTBL1 #1: 24h 1.93×10^{-12} (*), 3.72×10^{-11} (#), 3.92×10^{-9} ($), 48h 2.08×10^{-13} (*), 2.74×10^{-12} (#), 2.28×10^{-14} ($), 72h 2.56×10^{-15} (*), 1.44×10^{-16} (#), 1.07×10^{-17} ($), siTBL #2: 24h 7.60×10^{-13} (*), 1.21×10^{-11} (#), 8.86×10^{-10} ($), 48h 1.18×10^{-13} (*), 1.41×10^{-12} (#), 1.40×10^{-14} ($), 72h 1.41×10^{-15} (*), 8.84×10^{-17} (#), 1.11×10^{-17} ($); two-way ANOVA with Bonferroni post-test; p-values panel (B): siNC #2: 3.44×10^{-3}, siTBL1: 5.24×10^{-6} (*), 7.52×10^{-6} ($); one-way ANOVA with Bonferroni post-test. Data in (A) and (B) plotted as mean ± SEM.

(C) Protein expression of TBL1 and PI3 kinase p100α 24–72 h after siRNA-mediated knockdown. β-actin served as a loading control.

(D–E) Densitometric quantification of immunoblot shown in C, normalized to β-actin for TBL1 (D) and PI3K p110α (E).

Fig. S4. TBL1 depletion leads to reduced invasiveness

Matrigel invasion assay was performed with murine Panc02 cells with stable expression of shRNA (A) or human Capan-1 cells after transfection with siRNA (B). Invading cells per microscopy field were counted. n=8 (A) or n=4–6 (B) microscopy fields per group, significantly different ($p < 0.05$) from control (no siRNA or no shRNA) *, (siNC #1 or shNC) #, (siNC #2) $; p-values panel (A) 3.30×10^{-11} (*), 1.61×10^{-10} (#); p-values panel (B): siNC #1: 1.05×10^{-3} (*), siNC #2: 1.45×10^{-4} (#), siTBL1 #1: 5.94×10^{-8} (*), 3.34×10^{-11} (#), 6.01×10^{-6} ($), siTBL1 #2: 3.90×10^{-5} (*), 8.91×10^{-10} (#), 2.40×10^{-3} ($) ; one-way ANOVA with Bonferroni post-test.

All data plotted as mean ± SEM.

Fig. S5. TBL1 does not control mitochondrial oxidative phosphorylation

Mitochondrial function of siRNA-transfected Capan-1 measured by oxygen consumption rate (OCR) under basal conditions, after injection of the mitochondrial ATPase inhibitor oligomycin, the mitochondrial uncoupler FCCP, and the Complex I and Complex III inhibitors antimycin A and rotenone. n=8–10 cell culture wells per group; two-way ANOVA with Bonferroni-post test; no comparisons were significantly different. Data plotted as mean ± SEM.

Fig. S6. PI3 kinase protein expression is reduced in TBL1-deficient cells

(A) mRNA expression of CDK2, CDK4 and PIK3CA in siRNA-transfected Capan-1 cells. n=3 cell culture wells per group; significantly different ($p < 0.05$) from control (no

siRNA) *, (siNC #2) $; p-values: CDK2: 2.73×10^{-4} (*), 1.97×10^{-4} ($), CDK4: 1.88×10^{-4} (*), 1.13×10^{-3} ($), PIK3CA: 4.08×10^{-4} (*), 6.98×10^{-4} ($); one-way ANOVA with Bonferroni post-test. Data plotted as mean ± SEM

(B) Densitometric quantification of immunoblots shown in Fig. 3B, normalized to VCP.

(C) Relative mRNA expression of PI3 kinase subunits in Affymetrix gene expression microarray from siRNA-transfected Capan-1 cells. n=3 microarrays per group, $ p = $10^{-10.12}$ determined as described in Material and Methods.

Fig. S7. TBL1-depletion leads to changes in cell cycle

Cell cycle analysis of Capan-1 cells 48h after siRNA-mediated knockdown by propidium iodide staining and FACS measurement. n=3 cell culture wells per group; significantly different ($p < 0.05$) from control (no siRNA) *, (siNC #1) #, (siNC #2) $; p-values:
G0/G1 phase: siNC #1: 3.05×10^{-4} (*); siNC #2: 1.24×10^{-4} (#); siTBL1 #1: 1.43×10^{-7} (*), 3.10×10^{-5} (#), 9.07×10^{-8} ($); siTBL1 #2: 7.82×10^{-7} (*), 5.90×10^{-4} (#), 4.59×10^{-7} ($); S phase: siNC #1: 1.83×10^{-3} (*); siNC #2: 8.84×10^{-5} (#); siTBL1 #1: 2.32×10^{-6} (*), 6.50×10^{-4} (#), 3.95×10^{-7} ($); siTBL1 #2: 8.38×10^{-8} (*), 3.92×10^{-6} (#), 2.25×10^{-8} ($); G2 phase: siNC #1: 2.43×10^{-5} (*); siNC #2: 2.01×10^{-5} (*); siTBL1 #1: 3.48×10^{-6} (*); siTBL1 #2: 8.81×10^{-8} (*), 1.55×10^{-4} (#), 1.95×10^{-4} ($); one-way ANOVA with Bonferroni post-test. Data plotted as mean ± SEM.

Fig. S8. TBL1 binding site in the PIK3CA promoter region.

Peak region (red bar) for TBL1 binding to the PI3 kinase p110α catalytic subunit gene (PIK3CA) in mouse and human.

Fig. S9. PI3 kinase re-constitution in TBL1 siRNA-treated Panc02 cells.

Western blot analysis of control and TBL1 shRNA transfected Panc02 cells overexpressing PI3K using the indicated antibodies.

Fig. S10. TBL1-depletion slows tumor growth and enhances chemosensitivity

(A) Subcutaneous tumors treated with repeated injections of shRNA-carrying Adenovirus

(B) Subcutaneous tumors with stable shRNA expression after treatment with Gemcitabine (GEM) or vehicle (NaCl)

Fig. S11. Ki-67 expression is reduced in TBL1-deficient tumors

Ki-67 positive tumor area in paraffin-embedded tissue sections of subcutaneous Panc02 tumors with stable shRNA expression. n=4–5 tumors per group; * $p = 4.29\times10^{-3}$, two-way ANOVA with Bonferroni post-test. Data plotted as mean ± SEM

Fig. S12. TBL1-depletion leads to reduction of PI3 kinase p110α and cell-cycle-associated proteins

Densitometric quantification of immunoblots shown in Fig. 5, normalized to VCP. n=3 bands as seen in Fig. 5. significantly different $p < 0.05$ at indicated comparisons: overall shNC vs. shTBL1 (*), overall NaCl vs. GEM (#), shNC+GEM vs. shTBL1+GEM ($), shNC+NaCl vs. shNC+GEM (†), shTBL1+NaCl vs. shTBL1+GEM (‡); p-values: TBL1 2.52×10^{-5} (*); PI3K p110α 7.07×10^{-3}(*); PTEN 0.0208 (#), 0.0257 (‡); total Akt 5.76×10^{-4} (*); CDK2 0.0335 (#); total Gsk3β 2.21×10^{-3} (*), 6.79×10^{-3} ($), CDK4 6.10×10^{-3} (*), 0.0493 ($); two-way ANOVA with Bonferroni post-test. Data plotted as mean ± SEM.

Fig. S1

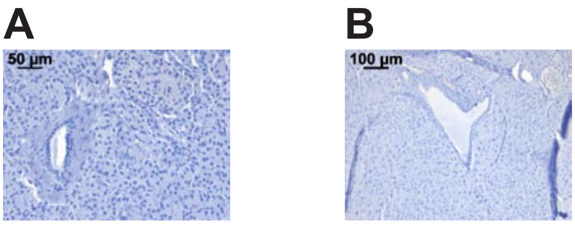

Fig. S2

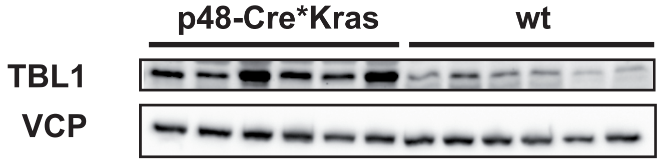

Fig. S3

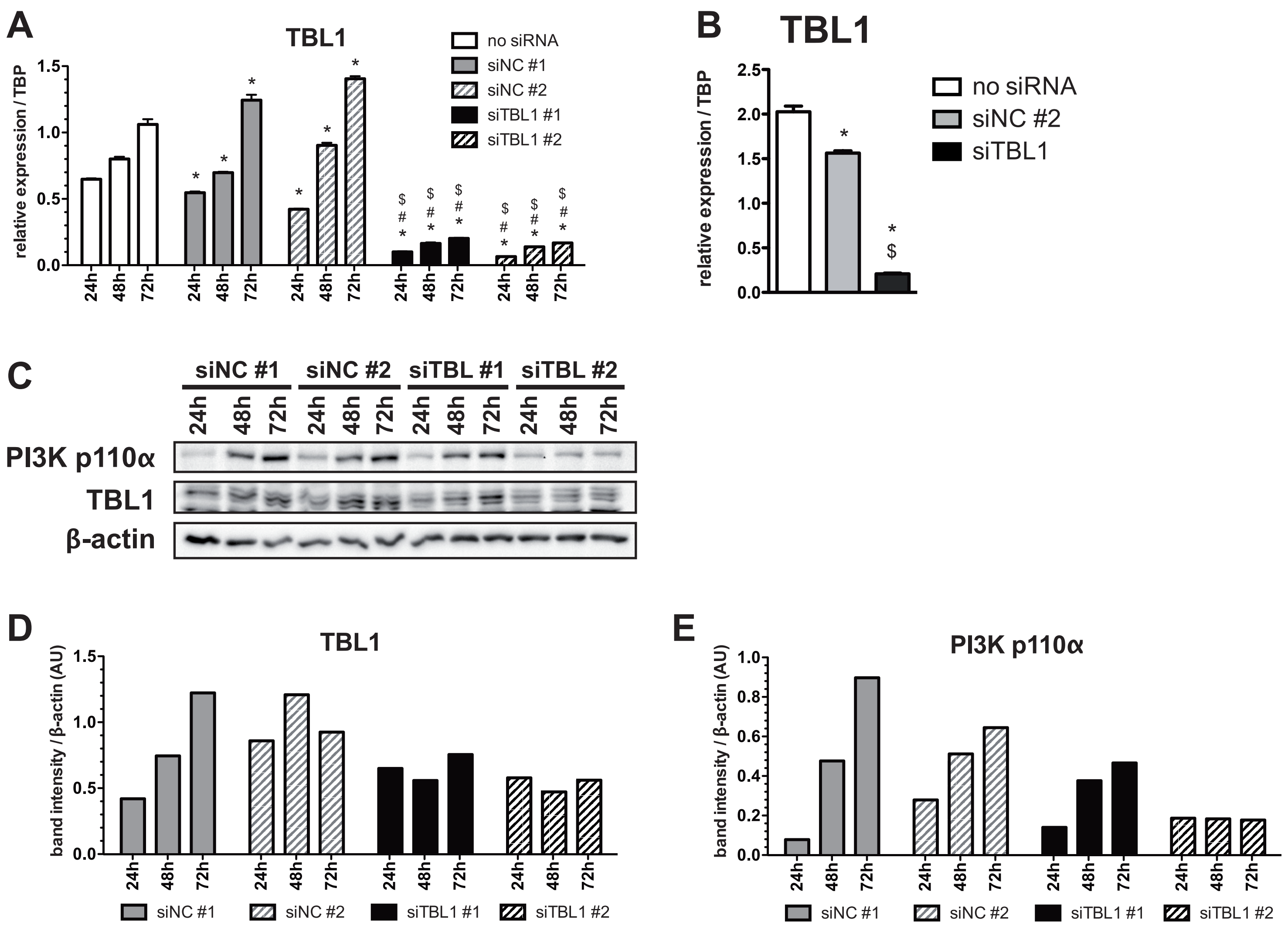
A
TBL1
relative expression / TBP
no siRNA
siNC #1
siNC #2
siTBL1 #1
siTBL1 #2
24h
48h
72h
B
TBL1
no siRNA
siNC #2
siTBL1
C
siNC #1
siNC #2
siTBL #1
siTBL #2
PI3K p110α
TBL1
β-actin
D
TBL1
band intensity / β-actin (AU)
E
PI3K p110α

Fig. S4

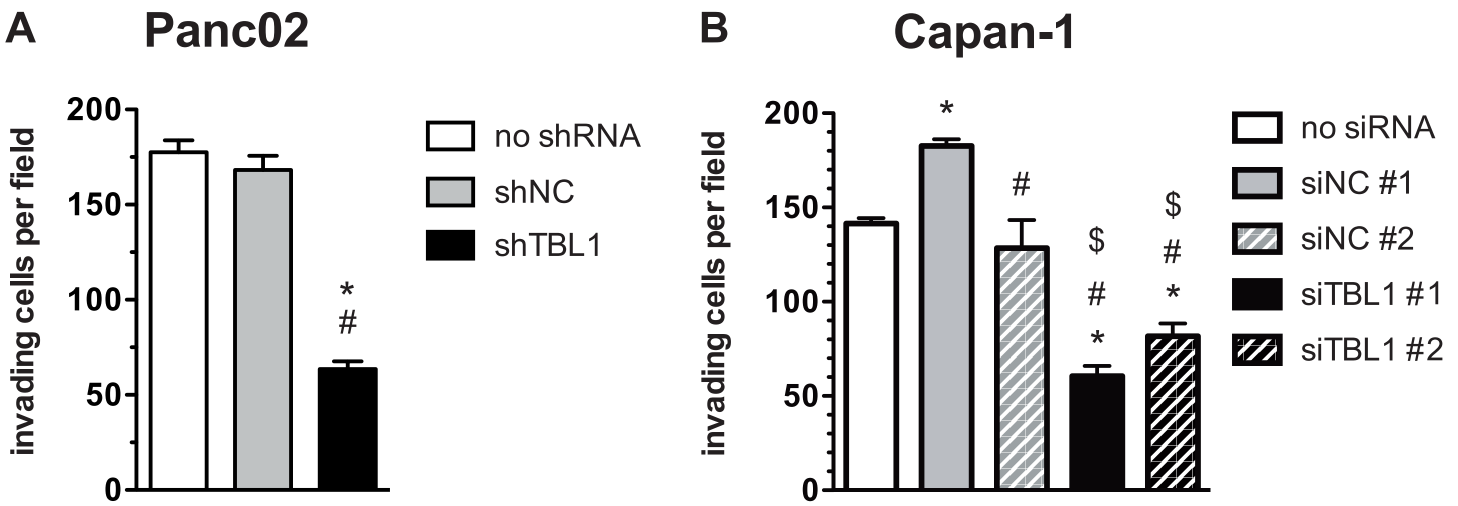
A
Panc02
invading cells per field
200
150
100
50
0
*
#
no shRNA
shNC
shTBL1
B
Capan-1
invading cells per field
200
150
100
50
0
*
#
$
#
*
$
#
*
no siRNA
siNC #1
siNC #2
siTBL1 #1
siTBL1 #2

Fig. S5

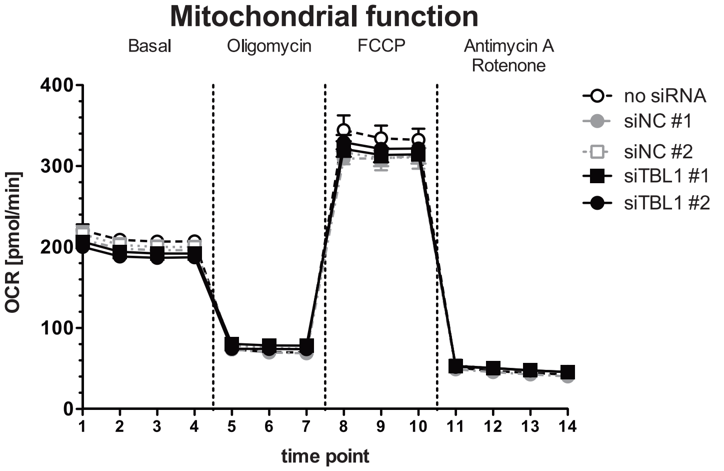
Mitochondrial function
Basal
Oligomycin
FCCP
Antimycin A
Rotenone
OCR [pmol/min]
0
100
200
300
400
1
2
3
4
5
6
7
8
9
10
11
12
13
14
time point
no siRNA
siNC #1
siNC #2
siTBL1 #1
siTBL1 #2

Fig. S6

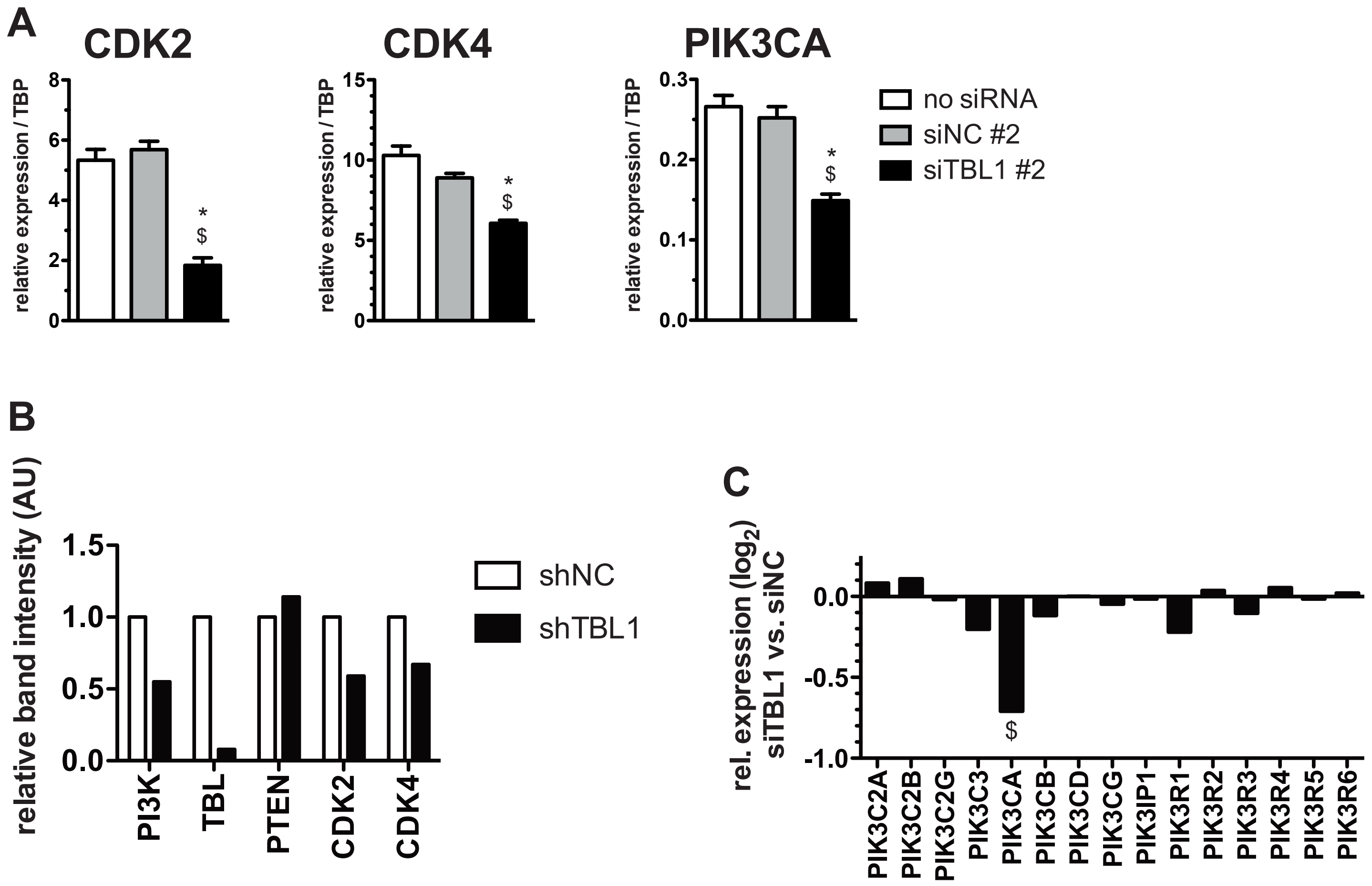
A
CDK2
CDK4
PIK3CA
relative expression / TBP
no siRNA
siNC #2
siTBL1 #2
B
relative band intensity (AU)
shNC
shTBL1
PI3K
TBL
PTEN
CDK2
CDK4
C
rel. expression (log_2)
siTBL1 vs. siNC
PIK3C2A
PIK3C2B
PIK3C2G
PIK3C3
PIK3CA
PIK3CB
PIK3CD
PIK3CG
PIK3IP1
PIK3R1
PIK3R2
PIK3R3
PIK3R4
PIK3R5
PIK3R6

Fig. S7

A

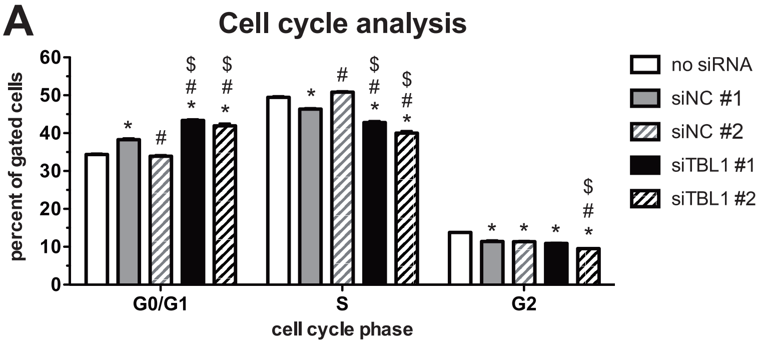

Cell cycle analysis
percent of gated cells
60
50
40
30
20
10
0
G0/G1
S
G2
cell cycle phase
no siRNA
siNC #1
siNC #2
siTBL1 #1
siTBL1 #2

Fig. S8

mouse chromosome 3: 32 396 859–32 397 027

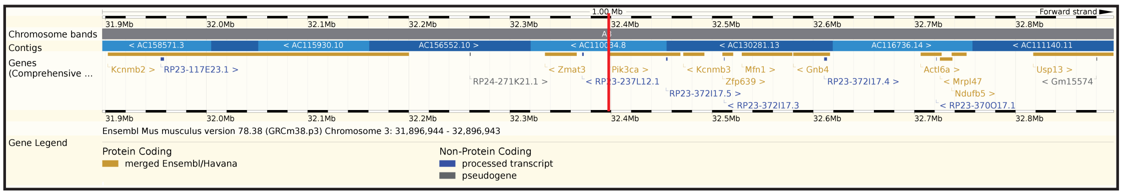

human chromosome 3: 179 147 899–179 148 101

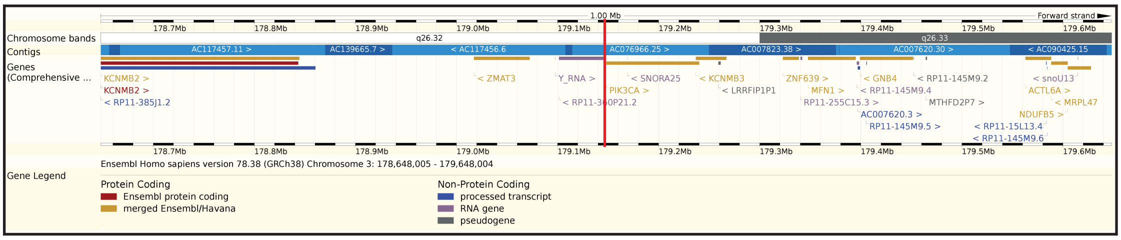

Fig. S9

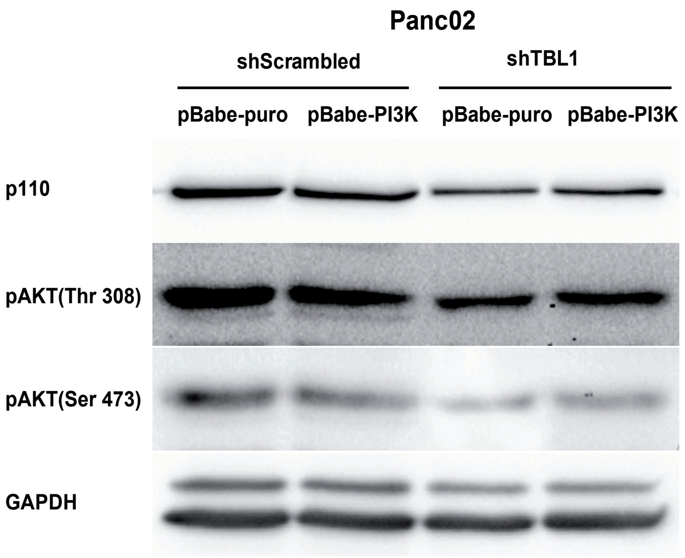

Panc02
shScrambled
shTBL1
pBabe-puro
pBabe-PI3K
pBabe-puro
pBabe-PI3K
p110
pAKT(Thr 308)
pAKT(Ser 473)
GAPDH

Fig. S10

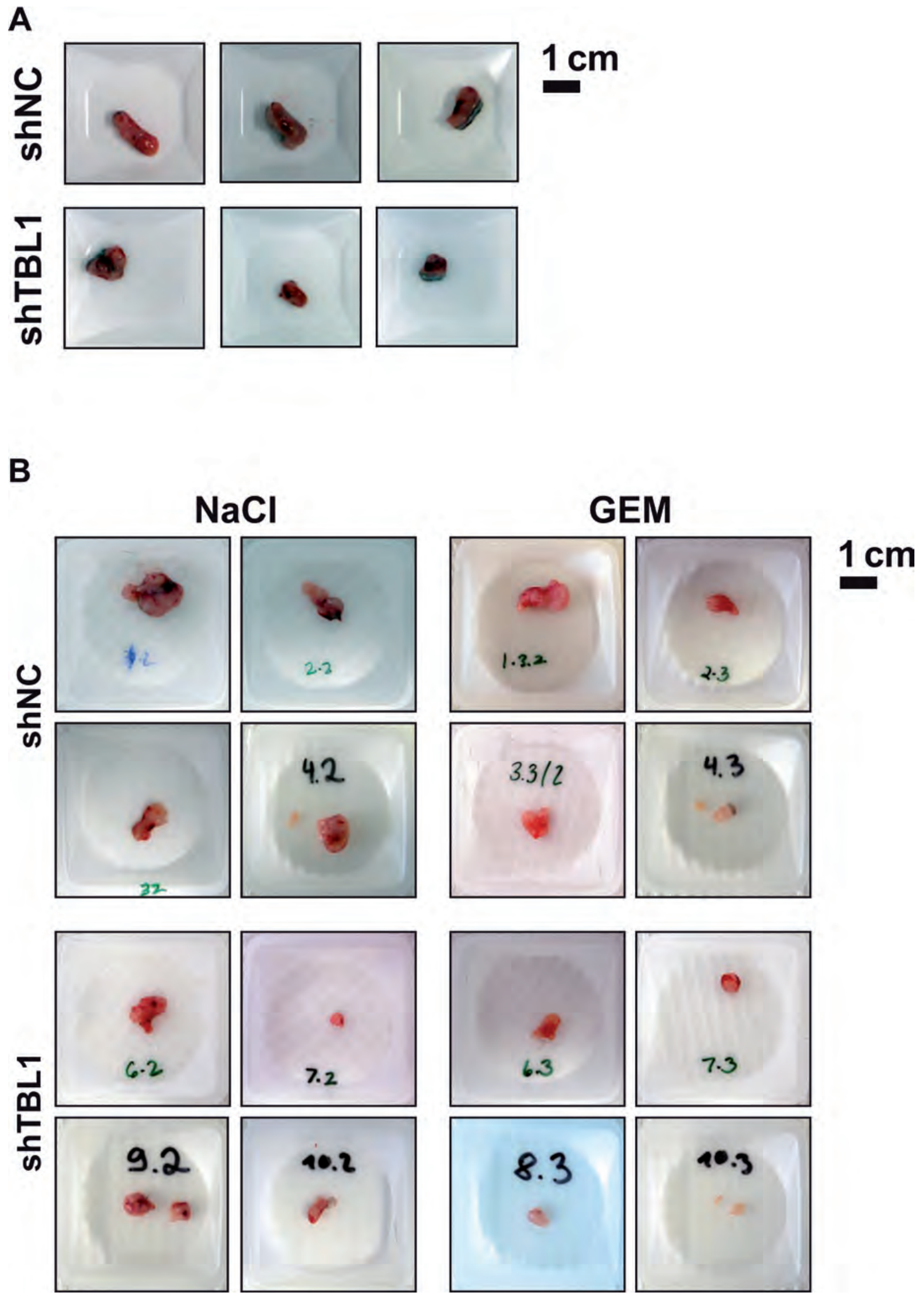

A
shNC
shTBL1
1 cm
B
NaCl
GEM
shNC
shTBL1
1 cm
2.2
1.3.2
2.3
4.2
3.3/2
4.3
6.2
7.2
6.3
7.3
9.2
10.2
8.3
10.3

Fig. S11

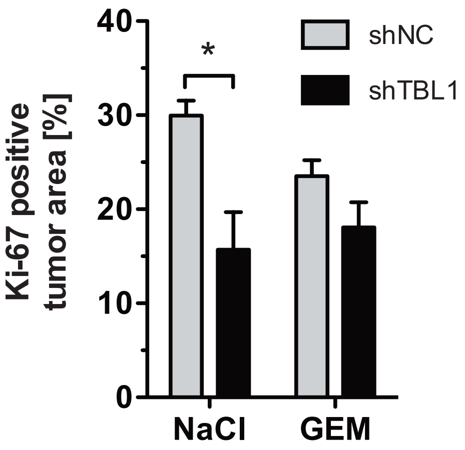

Fig. S12

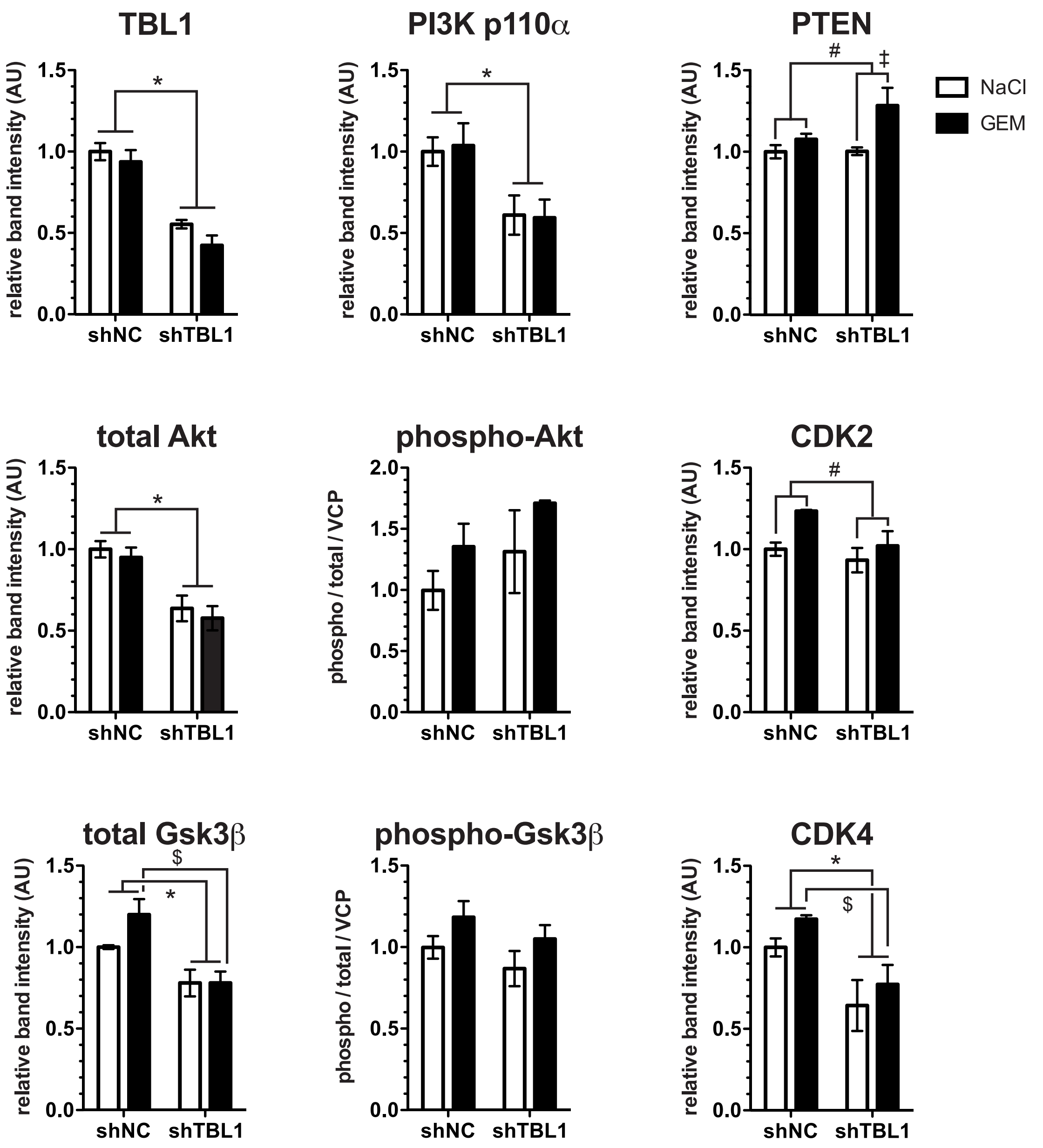
TBL1
PI3K p110α
PTEN
NaCl
GEM
total Akt
phospho-Akt
CDK2
total Gsk3β
phospho-Gsk3β
CDK4
relative band intensity (AU)
phospho / total / VCP
shNC
shTBL1
