# Supplementary material for: Transcriptional co-factor Transducin beta-like (TBL) 1 acts as a checkpoint in pancreatic cancer malignancy
Source: EMBO Mol Med. 2015 Jun 13;7(8):1048–62. doi: 10.15252/emmm.201404837 (PMC4551343; doi:10.15252/emmm.201404837)
Supplement: Supplementary file 3 [file emmm0007-1048-sd3.docx]

**Table S2: Wnt target genes are not significantly regulated by TBL1**

| **Gene Symbol** | **Name** | **fold change (log_2_)** | **–log_10_(*p*)** | **Significant  (–log_10_(*p*)>6.04)** |
| --- | --- | --- | --- | --- |
| ATOH1 | atonal homolog 1 | 0.043 | 0.493 | no |
| AXIN2 | axin 2 | –0.031 | 0.774 | no |
| BGLAP | osteocalcin | 0.045 | 0.590 | no |
| BIRC5 | baculoviral IAP repeat containing 5 | 0.176 | 5.759 | no |
| BMP2 | bone morphogenetic protein 2 | –0.019 | 0.273 | no |
| BMP4 | bone morphogenetic protein 4 | –0.075 | 1.528 | no |
| BTRC | beta-transducin repeat containing | –0.007 | 0.102 | no |
| CACNA1G | calcium channel, voltage-dependent, T type, alpha 1G subunit | –0.030 | 0.724 | no |
| CCND1 | cyclin D1 | 0.123 | 2.614 | no |
| CCND2 | cyclin D2 | –0.017 | 0.280 | no |
| CCND3 | cyclin D3 | 0.099 | 2.237 | no |
| CD44 | CD44 molecule | 0.095 | 3.286 | no |
| CDH1 | cadherin 1, type 1, E-cadherin | –0.387 | 9.483 | yes |
| CDKN2A | cyclin-dependent kinase inhibitor 2A (p16-Ink4a) | 0.051 | 1.385 | no |
| CDX1 | caudal type homeobox 1 | –0.034 | 0.374 | no |
| CDX4 | caudal type homeobox 4 | 0.029 | 0.355 | no |
| CLDN1 | claudin 1 | 0.622 | 10.396 | yes |
| CTLA4 | cytotoxic T-lymphocyte-associated protein 4 | 0.017 | 0.384 | no |
| CYR61 | cysteine-rich, angiogenic inducer, 61 | 0.363 | 7.886 | yes |
| DKK1 | dickkopf homolog 1 | 0.228 | 6.443 | yes |
| DKK2 | dickkopf homolog 2 | 0.016 | 0.217 | no |
| DKK3 | dickkopf homolog 3 | 0.020 | 0.302 | no |
| DKK4 | dickkopf homolog 4 | 0.000 | 0.001 | no |
| DKKL1 | dickkopf-like 1 | –0.057 | 1.163 | no |
| DLK1 | delta-like 1 homolog | 0.027 | 0.278 | no |
| DLL1 | delta-like 1 | 0.007 | 0.091 | no |
| EDA | ectodysplasin A | –0.032 | 0.679 | no |
| EDN1 | endothelin 1 | –0.253 | 7.187 | yes |
| EFNB1 | ephrin-B1 | –0.176 | 3.829 | no |
| EFNB2 | ephrin-B2 | –0.019 | 0.461 | no |
| EFNB3 | ephrin-B3 | –0.005 | 0.051 | no |
| EGFR | epidermal growth factor receptor | –0.156 | 5.208 | no |
| EGR1 | early growth response 1 | 0.056 | 0.975 | no |
| EGR2 | early growth response 2 | 0.031 | 0.327 | no |
| EGR3 | early growth response 3 | –0.008 | 0.095 | no |
| EGR4 | early growth response 4 | 0.005 | 0.050 | no |
| EMP1 | epithelial membrane protein 1 | 0.143 | 2.804 | no |
| EMP2 | epithelial membrane protein 2 | 0.054 | 1.835 | no |
| EMP3 | epithelial membrane protein 3 | 0.136 | 3.536 | no |
| EN1 | engrailed homeobox 1 | 0.019 | 0.264 | no |
| EN2 | engrailed homeobox 2 | 0.222 | 3.918 | no |
| ENPP2 | ectonucleotide pyrophosphatase/phosphodiesterase 2 | –0.002 | 0.016 | no |
| FGF4 | fibroblast growth factor 4 | –0.014 | 0.225 | no |
| FGF9 | fibroblast growth factor 9 | –0.015 | 0.207 | no |
| FGF18 | fibroblast growth factor 18 | –0.114 | 3.432 | no |
| FGF20 | fibroblast growth factor 20 | 0.000 | 0.001 | no |
| FN1 | fibronectin 1 | 0.024 | 0.610 | no |
| FOSL1 | FOS-like antigen 1 | –0.043 | 0.390 | no |
| FOXN1 | forkhead box N1 | 0.026 | 0.237 | no |
| FST | follistatin | 0.000 | 0.005 | no |
| FZD5 | frizzled homolog 5 | 0.383 | 6.630 | yes |
| FZD7 | frizzled homolog 7 | 0.172 | 4.612 | no |
| GAST | gastrin | 0.021 | 0.191 | no |
| GBX2 | gastrulation brain homeobox 2 | 0.071 | 1.680 | no |
| GCG | glucagon | –0.007 | 0.110 | no |
| GJA1 | gap junction protein, alpha 1, 43kDa | –0.065 | 1.508 | no |
| GJB6 | gap junction protein, beta 6, 30kDa | 0.031 | 0.851 | no |
| GREM1 | gremlin 1 | 0.004 | 0.057 | no |
| GREM2 | gremlin 2 | –0.028 | 0.858 | no |
| HOXA6 | homeobox A6 | 0.011 | 0.122 | no |
| HOXB6 | homeobox B6 | 0.006 | 0.054 | no |
| ID2 | inhibitor of DNA binding 2, dominant negative helix-loop-helix protein | 0.183 | 3.114 | no |
| IGF1 | insulin-like growth factor 1 | 0.008 | 0.142 | no |
| IGF2 | insulin-like growth factor 2 | n.a. | n.a. | n.a. |
| IL6 | interleukin 6 | –0.025 | 0.339 | no |
| IL8 | interleukin 8 | –0.230 | 3.955 | no |
| IRX3 | iroquois homeobox 3 | –0.025 | 0.411 | no |
| ISL1 | ISL LIM homeobox 1 | –0.213 | 5.553 | no |
| ISLR | immunoglobulin superfamily containing leucine-rich repeat | –0.003 | 0.035 | no |
| JAG1 | jagged 1 | 0.217 | 5.057 | no |
| JUN | jun proto-oncogene | –0.078 | 2.250 | no |
| KLF5 | Kruppel-like factor 5 | 0.101 | 2.661 | no |
| KRT1 | keratin 1 | –0.065 | 0.903 | no |
| KRT2 | keratin 2 | –0.026 | 0.232 | no |
| KRT3 | keratin 3 | –0.012 | 0.109 | no |
| KRT4 | keratin 4 | 0.033 | 0.507 | no |
| KRT5 | keratin 5 | 0.008 | 0.087 | no |
| KRT6A | keratin 6A | 0.013 | 0.114 | no |
| KRT6B | keratin 6B | 0.010 | 0.165 | no |
| KRT6C | keratin 6C | 0.044 | 0.408 | no |
| KRT7 | keratin 7 | –0.057 | 0.868 | no |
| KRT9 | keratin 9 | 0.019 | 0.133 | no |
| KRT10 | keratin 10 | –0.020 | 0.240 | no |
| KRT12 | keratin 12 | 0.046 | 0.915 | no |
| KRT13 | keratin 13 | 0.018 | 0.289 | no |
| KRT14 | keratin 14 | –0.024 | 0.129 | no |
| KRT15 | keratin 15 | –0.342 | 7.264 | yes |
| KRT16 | keratin 16 | –0.088 | 1.716 | no |
| KRT17 | keratin 17 | 0.004 | 0.028 | no |
| KRT19 | keratin 19 | –0.313 | 8.541 | yes |
| KRT20 | keratin 20 | 0.040 | 0.647 | no |
| KRT23 | keratin 23 (histone deacetylase inducible) | –0.173 | 2.573 | no |
| KRT24 | keratin 24 | –0.020 | 0.279 | no |
| KRT25 | keratin 25 | –0.036 | 0.524 | no |
| KRT27 | keratin 27 | –0.033 | 0.418 | no |
| KRT31 | keratin 31 | 0.007 | 0.048 | no |
| KRT32 | keratin 32 | –0.009 | 0.083 | no |
| KRT33B | keratin 33B | 0.020 | 0.225 | no |
| KRT34 | keratin 34 | 0.054 | 0.810 | no |
| KRT35 | keratin 35 | 0.071 | 1.898 | no |
| KRT36 | keratin 36 | –0.002 | 0.024 | no |
| KRT37 | keratin 37 | 0.023 | 0.456 | no |
| KRT38 | keratin 38 | –0.066 | 0.797 | no |
| KRT40 | keratin 40 | –0.001 | 0.007 | no |
| KRT71 | keratin 71 | 0.027 | 0.182 | no |
| KRT72 | keratin 72 | –0.018 | 0.212 | no |
| KRT73 | keratin 73 | 0.048 | 0.900 | no |
| KRT74 | keratin 74 | –0.030 | 0.659 | no |
| KRT75 | keratin 75 | –0.001 | 0.021 | no |
| KRT76 | keratin 76 | 0.056 | 0.749 | no |
| KRT77 | keratin 77 | 0.030 | 0.409 | no |
| KRT78 | keratin 78 | 0.011 | 0.122 | no |
| KRT79 | keratin 79 | –0.065 | 1.431 | no |
| KRT80 | keratin 80 | –0.268 | 6.764 | yes |
| KRT81 | keratin 81 | 0.006 | 0.047 | no |
| KRT82 | keratin 82 | 0.010 | 0.123 | no |
| KRT83 | keratin 83 | 0.004 | 0.045 | no |
| KRT84 | keratin 84 | –0.143 | 1.912 | no |
| KRT85 | keratin 85 | –0.017 | 0.265 | no |
| KRT86 | keratin 86 | –0.049 | 0.444 | no |
| KRT222 | keratin 222 | 0.047 | 1.437 | no |
| L1CAM | L1 cell adhesion molecule | 0.007 | 0.114 | no |
| LBH | limb bud and heart development homolog | 0.034 | 0.384 | no |
| LEF1 | lymphoid enhancer-binding factor 1 | 0.025 | 0.899 | no |
| LGR5 | leucine-rich repeat containing G protein-coupled receptor 5 | –0.097 | 1.407 | no |
| MET | met proto-oncogene (hepatocyte growth factor receptor) | –0.174 | 3.964 | no |
| MITF | microphthalmia-associated transcription factor | 0.061 | 1.100 | no |
| MMP2 | matrix metallopeptidase 2 (gelatinase A) | 0.008 | 0.062 | no |
| MMP3 | matrix metallopeptidase 3 (stromelysin 1, progelatinase) | 0.090 | 2.091 | no |
| MMP7 | matrix metallopeptidase 7 (matrilysin, uterine) | 0.027 | 0.505 | no |
| MMP9 | matrix metallopeptidase 9 (gelatinase B, 92kDa gelatinase, 92kDa type IV collagenase) | –0.087 | 1.152 | no |
| MMP10 | matrix metallopeptidase 10 (stromelysin 2) | –0.035 | 0.335 | no |
| MMP11 | matrix metallopeptidase 11 (stromelysin 3) | 0.015 | 0.219 | no |
| MMP26 | matrix metallopeptidase 26 | 0.050 | 0.578 | no |
| MSL1 | male-specific lethal 1 homolog | –0.052 | 1.278 | no |
| MYC | v-myc myelocytomatosis viral oncogene homolog | 0.134 | 3.321 | no |
| MYCBP | c-myc binding protein | –0.030 | 0.477 | no |
| MYCN | v-myc myelocytomatosis viral related oncogene, neuroblastoma derived | 0.026 | 0.638 | no |
| MYF6 | myogenic factor 6 (herculin) | 0.004 | 0.048 | no |
| NANOG | Nanog homeobox | n.a. | n.a. | n.a. |
| NEUROD1 | neurogenic differentiation 1 | 0.003 | 0.048 | no |
| NEUROG1 | neurogenin 1 | 0.003 | 0.041 | no |
| NKX2-2 | NK2 homeobox 2 | –0.039 | 0.431 | no |
| NLK | nemo-like kinase | –0.004 | 0.043 | no |
| NOS2 | nitric oxide synthase 2, inducible | 0.027 | 0.222 | no |
| NRCAM | neuronal cell adhesion molecule | –0.026 | 0.447 | no |
| OVOL1 | ovo-like 1 | –0.083 | 2.236 | no |
| PITX2 | paired-like homeodomain 2 | 0.090 | 1.889 | no |
| PLAUR | plasminogen activator, urokinase receptor | –0.013 | 0.238 | no |
| POSTN | periostin, osteoblast specific factor | –0.043 | 1.260 | no |
| POU5F1 | POU class 5 homeobox 1 | n.a. | n.a. | n.a. |
| PPARD | peroxisome proliferator-activated receptor delta | –0.034 | 0.863 | no |
| PRL | prolactin | –0.080 | 1.181 | no |
| PRL | prolactin | –0.080 | 1.181 | no |
| PTGS2 | prostaglandin-endoperoxide synthase 2 (cyclooxygenase-2) | –0.031 | 0.580 | no |
| PTTG1 | pituitary tumor-transforming 1 | –0.024 | 0.415 | no |
| PTTG2 | pituitary tumor-transforming 2 | 0.058 | 0.984 | no |
| RARG | retinoic acid receptor, gamma | –0.030 | 0.486 | no |
| RET | ret proto-oncogene | –0.012 | 0.212 | no |
| RHOU | ras homolog gene family, member U | 0.040 | 1.362 | no |
| RUNX2 | runt-related transcription factor 2 | 0.055 | 1.935 | no |
| SALL4 | sal-like 4 (Drosophila) | –0.053 | 1.560 | no |
| SFRP2 | secreted frizzled-related protein 2 | –0.008 | 0.132 | no |
| SIX3 | SIX homeobox 3 | 0.008 | 0.110 | no |
| SNAI1 | snail homolog 1 (Drosophila) | –0.037 | 0.337 | no |
| SOX2 | SRY (sex determining region Y)-box 2 | 0.002 | 0.021 | no |
| SOX9 | SRY (sex determining region Y)-box 9 | –0.111 | 3.428 | no |
| SOX17 | SRY (sex determining region Y)-box 17 | –0.002 | 0.025 | no |
| SP5 | Sp5 transcription factor | 0.021 | 0.248 | no |
| STRA6 | stimulated by retinoic acid gene 6 homolog (mouse) | 0.014 | 0.270 | no |
| T | T, brachyury homolog (mouse) | –0.054 | 0.764 | no |
| TBC1D9 | TBC1 domain family, member 9 (with GRAM domain) | –0.147 | 3.895 | no |
| TCF4 | transcription factor 4 | –0.048 | 1.902 | no |
| TCF7 | transcription factor 7 (T-cell specific, HMG-box) | 0.063 | 1.597 | no |
| TERT | telomerase reverse transcriptase | –0.028 | 0.784 | no |
| TIAM1 | T-cell lymphoma invasion and metastasis 1 | 0.038 | 0.922 | no |
| TNFRSF19 | tumor necrosis factor receptor superfamily, member 19 | 0.012 | 0.210 | no |
| TNFSF9 | tumor necrosis factor (ligand) superfamily, member 9 | 0.196 | 3.523 | no |
| TNFSF11 | tumor necrosis factor (ligand) superfamily, member 11 | –0.010 | 0.207 | no |
| TWIST1 | twist homolog 1 (Drosophila) | 0.012 | 0.135 | no |
| TWIST2 | twist homolog 2 (Drosophila) | –0.041 | 0.530 | no |
| VCAN | versican | 0.049 | 1.364 | no |
| VEGFA | vascular endothelial growth factor A | 0.083 | 2.748 | no |
| VEGFB | vascular endothelial growth factor B | 0.024 | 0.468 | no |
| VEGFC | vascular endothelial growth factor C | 0.035 | 0.392 | no |
| WISP1 | WNT1 inducible signaling pathway protein 1 | –0.039 | 0.500 | no |
| WISP2 | WNT1 inducible signaling pathway protein 2 | –0.007 | 0.056 | no |
| WISP3 | WNT1 inducible signaling pathway protein 3 | 0.021 | 0.304 | no |
| WNT1 | wingless-type MMTV integration site family, member 1 | 0.052 | 0.721 | no |
| WNT3A | wingless-type MMTV integration site family, member 3A | n.a. | n.a. | n.a |

190 genes

4 not present on AffyChip

= 186 evaluated

9 significant

Two-sided Chi square test

| Chi square | 0.003133 |
| --- | --- |
| degrees of freedom | 1 |
| p-value | 0.9554 |

|  | Wnt-target | no Wnt target | Σ |
| --- | --- | --- | --- |
| Significant | 9 | 876 | 885 |
| not significant | 177 | 17946 | 18123 |
| Σ | 186 | 18822 | 19008 |
