# Supplementary material for: Transcriptional co-factor Transducin beta-like (TBL) 1 acts as a checkpoint in pancreatic cancer malignancy
Source: EMBO Mol Med. 2015 Jun 13;7(8):1048–62. doi: 10.15252/emmm.201404837 (PMC4551343; doi:10.15252/emmm.201404837)
Supplement: Supplementary file 4 [file emmm0007-1048-sd4.pdf]

EMBO Molecular Medicine

Transcriptional co-factor Transducin beta-like (TBL) 1 acts as a checkpoint in pancreatic cancer malignancy

Christian Stoy, Aishwarya Sundaram, Marcos Rios Garcia, Xiaoyue Wang, Oksana Seibert, Annika Zota, Susann Wendler, David Männle, Ulf Hinz, Carsten Sticht, Maria Muciek, Norbert Gretz, Adam Rose, Vera Greiner, Thomas Hofmann, Andrea Bauer, Jörg Hoheisel, Mauricio Berriel Diaz, Matthias M. Gaida, Jens Werner, Tobias Schafmeier, Oliver Strobel and Stephan Herzig

Corresponding author: Stephan Herzig, DKFZ, ZMBH

Review timeline:

Submission date:	11 November 2014
Editorial Decision:	18 December 2014
Revision received:	03 March 2015
Editorial Decision:	23 March 2015
Revision received:	21 April 2015
Editorial Decision:	28 April 2015
Revision received:	13 May 2015
Accepted:	15 May 2015

Transaction Report:

(Note: With the exception of the correction of typographical or spelling errors that could be a source of ambiguity, letters and reports are not edited. The original formatting of letters and referee reports may not be reflected in this compilation.)

Editor: Dr. Buccione

1st Editorial Decision 18 December 2014

Thank you for the submission of your manuscript to EMBO Molecular Medicine. We are sorry that it has taken longer than we would have liked to get back to you on your manuscript. We have now heard back from the three Reviewers whom we asked to evaluate your manuscript.

In this case we experienced some difficulties in securing three competent and willing Reviewers, and further to this, two evaluations were delivered with delay.

You will see while the Reviewers are generally supportive of your work, all three express significant and to some extent, overlapping concerns that prevent us from considering publication at this time.

Reviewer 1 notes the lack of appropriate controls in the TBL1 knockdown experiments, which currently compromises their conclusiveness. S/he also lists a number of other concerns that require your action.

Reviewer 2 is more reserved and points to a number of experimental issues that require your action. S/he is also quite worried that the TBL1 knockdown data are poorly controlled and suggests appropriate rescue experiments, in addition to testing additional siRNAs. Reviewer 2 also feels that direct evidence is required to support the assertion that perturbation of PI3K signalling might underlie the effects of TBL1 silencing. This Reviewer, in addition to other points, also notes missing

controls in the ChIP experiments.

Reviewer 3 is of the opinion that metabolic consequences of TBL1 inhibition, and its effectors, need to be better analysed to support the claims. S/he also mentions the lack of appropriate controls in the ChIP experiments and the need to better establish the role of Pi3K.

Considered all the above, while publication of the paper cannot be considered at this stage, we would be pleased to consider revised submission, with the understanding that the Reviewers' concerns must be fully addressed, with additional experimental data where appropriate and that acceptance of the manuscript will entail a second round of review.

We would like to remind you that it is EMBO Molecular Medicine policy to allow a single round of revision only and that, therefore, acceptance or rejection of the manuscript will depend on the completeness of your responses included in the next, final version of the manuscript.

As you know, EMBO Molecular Medicine has a "scooping protection" policy, whereby similar findings that are published by others during review or revision are not a criterion for rejection. However, I do ask you to get in touch with us after three months if you have not completed your revision, to update us on the status. Please also contact us as soon as possible if similar work is published elsewhere.

Please note that EMBO Molecular Medicine now requires a complete author checklist (http://embomolmed.embopress.org/authorguide#editorial3) to be submitted with all revised manuscripts. Provision of the author checklist is mandatory at revision stage; the checklist is designed to enhance and standardize reporting of key information in research papers and to support reanalysis and repetition of experiments by the community. The list covers key information for figure panels and captions and focuses on statistics, the reporting of reagents, animal models and human subject-derived data, as well as guidance to optimise data accessibility. I am attaching the checklist here for your convenience. Should you have problems opening the attachment, please download it using the above link.

I look forward to seeing a revised form of your manuscript as soon as possible.

***** Reviewer's comments *****

Referee #1 (Remarks):

In this manuscript, Stoy and colleagues identified TBL1 as a critical regulator of PI3K signaling in human and murine pancreatic ductal adenocarcinoma (PDAC). They show that TBL1 expression increases during pancreatic carcinogenesis and correlates with shortened survival of PDAC patients. Mechanistically, TBL1 increases PDAC cell proliferation and invasiveness by the transcriptional regulation of PIK3CA. Importantly, the authors show that siRNA mediated knock-down of TBL1 delays tumor progression and increases chemosensitivity towards Gemcitabine in vivo.

This is a very interesting and well conducted study that uses several different pancreatic cancer models in vitro and in vivo. The data are compelling and sound. However, there are some concerns and suggestions that should be taken into account.

Specific comments:

1. Results page 6: Which cell type is meant by "preneoplastic epithelial cells" (line 14)? PanIN are defined as neoplastic cells.

2. A pathologist experienced in human and mouse PDAC should review the histological specimens shown in the manuscript.

3. The authors use a single siRNA to demonstrate that TBL1 knockdown impairs cell proliferation, glycolytic function and PIK3CA expression in the in vitro experiments. Because it has been shown

that siRNAs have profound off target effects, the authors should use at least a second siRNA targeting TBL1 to confirm their findings.

4. Fig. E4B: The significant reduction in invasion of control siRNA transfeted Capan1 cells vs. untransfected cells is of concern and argues for off target effects of the control siRNA.

5. Fig. 3B. Reduced expression of CDK2 upon TBL1 knockdown as suggested by the authors is not convincing.

6. Fig. 3E: It remains unclear how the ChIP data were normalized. A negative control should be included in the ChIP experiments.

7. The authors state that Wnt target gene expression is not altered by TBL1 knockdown, but don't show the data. The paper would be greatly strengthened if this data were shown.

8. A statement regarding approval of animal experiments is missing in the manuscript.

9. AsPC-1 and BxPC3 cells are listed in the methods section, but no data are shown.

Referee #2 (Remarks):

The Manuscript "Transcription co-factor Transducin beta-like (TBL)1 acts as a checkpoint in pancreatic cancer malignancy" by Stoy C. et al., tries to find out which is the relationship between TBL1 expression and pancreatic cancer onset and progression. Their hypothesis is based on the evidence that this transcriptional co-activator is involved in pathophysiological events of metabolically active tissues and Pancreatic Ductal Adenocarcinoma (PDAC) is tightly associated with metabolic dysfunction. Essentially they find a correlation between TBL1 expression and some aspects of pancreatic cancer cell proliferation, metabolism and invasion. Transcriptomic and ChiP-seq analyses allowed to determine a series of potential target genes, one of which, PI3K p110 subunit was validated as a direct target gene. In human pancreatic cancer patients they find correlation between TLB1 expression and survival, and in mice they find that silencing of TLB1 is decreasing pancreatic cancer growth as well as sensitizing to gemcitabine treatment in vivo.

As the authors themselves suggest, TLB1 might mediate proliferative and metabolic cues determining aggressive pancreatic cancer growth and chemo-resistance, possibly through regulation of the PI3K/Akt pathway. The paper is hence of great potential, however, all the statements throughout the paper remain correlative in nature. Moreover, an important novel finding is the observation that TBL1 mRNA and protein are upregulated only in the tumour, but what might control TBL1 in this context has not been addressed.
While the data of Fig. 4 are quite convincing (tumour growth and chemo-resistance studies in subcutaneously injected mice), all other results are either incomplete or confusing, as detailed in the point-by point comments here below. In general, to confirm some of the obtained results -that were performed exclusively with silencing of TBL1- at least some of them should be improved i) by using another siRNA and ii) with rescue experiments transferring a TBL1 expression vector which is resistant to the silencing. Moreover, if the authors believe that some of the phenotypes elicited by TBL1 silencing are due to the consequent alteration of glucose uptake or PI3K signalling or both, they should try to demonstrate this by recovering the siTBL1 phenotypes for example by overexpressing PI3K pathway components.

Major comments:

Fig. 1B is quite misleading for more than one reason: i) first of all normal and tumour tissues are shown with different magnifications (80 versus 25 or 50 micrometers); ii) unequal sections are

shown, since the section of normal pancreas (1) derives from an oblique cut of the acini, while in PanIn-1-3 (2-5) longitudinal sections are shown; iii) how many normal and tumour tissues were analysed by IHC?

Fig. 1D: same comment as for Fig. 1B

Fig. 2 in general: It seems that only one siRNA sequence was used, or was shRNA sequence different? This is not clear at all. In any case a different sequence for silencing must be added as a control. Moreover, Fig. E3 shows the efficiency of silencing at the mRNA level, but also a Western blot analysis should be added.

Fig. E4B: why in Capan-1 cells also siNC is affecting invasion in a manner similar to siTBL1?

Fig. 3C: Explanation of NaCl/GEM or triplicate loading is missing. (One has to arrive at the end of the paper to understand what is GEM). Moreover, some samples, like the first lane of shTBL1/NaCl do not correspond to what was stated in the text, e.g. PTEN and total Akt levels levels are lower than in control cells. Why not showing a representative Western blot and adding a protein density graph with the average {plus minus} st.dev. of all samples? This would be much easier to understand.

Fig. 3E: ChIP-PCR analysis lacks the control of TBL1 silencing and PCR of an other region in the promoter, different from the peaks in Fig. 3D.

Minor comments:

Fig. 1C: In the text (pg. 6) related to Fig. 1C, it is not clear why "genome-wide transcriptome analysis" is necessary to cite in this context, since only two genes (TBL1 and PNLIPRP2) were used in this analysis.

Fig. 2A, 2B: proliferation defects following TBL1 silencing go from 5% to 30% decrease, depending on species and assay, which is not so much to justify the sentence "..underlining the conclusion that TBL1 fulfills a conserved growth regulatory function..." which is overstated both because of the result and because other experiments should be added to demonstrate this function (e.g. Cell cycle FACS analyses, Western blot of Cell cycle markers etc.).

Again, at the end of the paragraph in pg. 7 the statement "..overall suggesting that TBL1 represents an important checkpoint in the tumorigenic program.." is again excessive in view of the confusing results in Fig. E4 and of the type of analyses that have been done till this point that are mainly correlations.

Referee #3 (Remarks):

In this paper, the authors show increased expression of TBL1 in human and mouse pancreatic cancers. They correlated TBL1 expression with poor survival in these patients. In vitro and in vivo experiments prove that TBL1 genetic inhibition results in decreased cell proliferation and tumor formation in mice. Finally, the authors show that the effects of TBL1 are mediated by the regulation of the expression of p110 Pi3K in tumor cells. Moreover, the authors claim that the effects of TBL1 are also mediated by the regulation of the metabolic switch in pancreatic cancer cells. The manuscript is well organized, and the experiments are clean. Data is of good quality. This reviewer has, however some major concerns about the mechanisms underlying TBL1 effects. In particular, I have the following concerns:

1. Differences in fig. 2B-C, although significant are very minor and cannot explain major effects on tumor progression.

2. The authors suggest that TBL1 couples proliferative, invasive, and metabolic pathways in pancreatic cancer. This is far to be proved and is only based in a single observation. How is the expression of genes involved in metabolic control changed upon TBL1 inhibition? What are the

metabolic effects of TBL1 overexpression in cancer cells? How is glutamine metabolism affected? Is there a major contribution of TBL1 on the metabolic control in normal cells?

3. ChIP-seq data analysis is required to understand the whole picture of the participation of TBL1 in these cancer cells. This should be provided. Are there other genes involved in metabolic control that are regulated by TBL1? Or the metabolic effects are also mediated by PI3K? These issues should be addressed.

4. The authors focus on PI3K p110 as a major target of TBL1. Promoter studies showing an effect of TBL1 on PI3K promoter activity would very well complement ChIP data.

5. The mechanisms of regulation of TBL1 expression are still poorly demonstrated. TBL1 is a transcriptional cofactor that does not have intrinsic transcriptional activity. The question here is: what is the major transcription factor associated to TBL1 in the PI3K promoter? It would very interesting to test if inhibition of this transcription factor would have the same effects on tumor progression.

6. Does PI3K overexpression in pancreatic cancer cells rescue proliferation and tumor formation?

1st Revision - authors' response 03 March 2015

We thank the referees for their positive and constructive comments. Please find our point-by-point response below.

Referee #1 (Remarks):

In this manuscript, Stoy and colleagues identified TBL1 as a critical regulator of PI3K signaling in human and murine pancreatic ductal adenocarcinoma (PDAC). They show that TBL1 expression increases during pancreatic carcinogenesis and correlates with shortened survival of PDAC patients. Mechanistically, TBL1 increases PDAC cell proliferation and invasiveness by the transcriptional regulation of PIK3CA. Importantly, the authors show that siRNA mediated knock-down of TBL1 delays tumor progression and increases chemosensitivity towards Gemcitabine in vivo.

This is a very interesting and well conducted study that uses several different pancreatic cancer models in vitro and in vivo. The data are compelling and sound. However, there are some concerns and suggestions that should be taken into account.

Specific comments:

1. Results page 6: Which cell type is meant by "preneoplastic epithelial cells" (line 14)? PanIN are defined as neoplastic cells.

We apologize for this confusing statement and have corrected the text accordingly.

2. A pathologist experienced in human and mouse PDAC should review the histological specimens shown in the manuscript.

As requested, a trained pathologist (Matthias M. Gaida, see updated author list) has re-analyzed the histological specimens and verified our statements. In addition, we have generated new figures now using identical magnifications and zoom-in panels throughout all figure panels (New Fig. 1C and 1D).

3. The authors use a single siRNA to demonstrate that TBL1 knockdown impairs cell proliferation, glycolytic function and PIK3CA expression in the in vitro experiments. Because it has been shown that siRNAs have profound off target effects, the authors should use at least a second siRNA targeting TBL1 to confirm their findings.

As requested, we have now used 1 additional control and 1 additional TBL1-specific siRNA to validate our initial findings. As shown in New Fig. 2A, 2B, 2D and E5, both siRNA constructs caused similar effects on cellular proliferation, glycolytic activity and respiratory capacity, thereby verifying our initial results.

4. Fig. E4B: The significant reduction in invasion of control siRNA transfeted Capan1 cells vs. untransfected cells is of concern and argues for off target effects of the control siRNA.

To validate our initial findings, we have used additional control and TBL1-specific siRNA constructs as outlined above. As shown in a New Fig. E4B also the second TBL1 siRNA inhibited the invasive behavior of pancreatic tumor cells, while the second control siRNA did not cause any obvious changes as compared with non-transfected cells.

5. Fig. 3B. Reduced expression of CDK2 upon TBL1 knockdown as suggested by the authors is not convincing.

We have quantified the TBL1-mediated down-regulation of CDK2 by densitometric analysis as shown in a New Fig. E6B. This quantification demonstrated an almost 2-fold down-regulation of CDK2 expression upon TBL1 siRNA treatment.

6. Fig. 3E: It remains unclear how the ChIP data were normalized. A negative control should be included in the ChIP experiments.

We apologize for the missing clarity in this point. We have now repeated all ChIP experiments by including not only non-specific IgG as a negative control but also TBL1 knockdown cells as well as a negative control PCR primer set targeting an unrelated region within the PIK3CA coding sequence. As shown in a New Fig. 3C, these experiments validated our initial findings and confirmed the specificity of the observed TBL1 recruitment to the PI3K locus. All results are shown as percentage of input material.

7. The authors state that Wnt target gene expression is not altered by TBL1 knockdown, but don't show the data. The paper would be greatly strengthened if this data were shown.

As requested, we have now compiled a New Supplemental Table E2 showing the absence of significant effects of TBL1 knockdown on known Wnt target genes.

8. A statement regarding approval of animal experiments is missing in the manuscript.

This has been corrected.

9. AsPC-1 and BxPC3 cells are listed in the methods section, but no data are shown.

This has been corrected.

Referee #2 (Remarks):

The Manuscript "Transcription co-factor Transducin beta-like (TBL)1 acts as a checkpoint in pancreatic cancer malignancy" by Stoy C. et al., tries to find out which is the relationship between TBL1 expression and pancreatic cancer onset and progression. Their hypothesis is based on the evidence that this transcriptional co-activator is involved in pathophysiological events of metabolically active tissues and Pancreatic Ductal Adenocarcinoma (PDAC) is tightly associated with metabolic dysfunction. Essentially they find a correlation between TBL1 expression and some aspects of pancreatic cancer cell proliferation, metabolism and invasion. Transcriptomic and ChiP-seq analyses allowed to determine a series of potential target genes, one of which, PI3K p110 subunit was validated as a direct target gene. In human pancreatic cancer patients they find correlation between TLB1 expression and survival, and in mice they find that silencing of TLB1 is decreasing pancreatic cancer growth as well as sensitizing to gemcitabine treatment in vivo.

As the authors themselves suggest, TLB1 might mediate proliferative and metabolic cues determining aggressive pancreatic cancer growth and chemo-resistance, possibly through regulation of the PI3K/Akt pathway. The paper is hence of great potential, however, all the statements throughout the paper remain correlative in nature. Moreover, an important novel finding is the observation that TBL1 mRNA and protein are upregulated only in the tumour, but what might control TBL1 in this context has not been addressed.

We thank the referee for bringing up this interesting issue. As TBL1 is known to act downstream of metabolic/endocrine signals in other tissues (e.g. Kulozik et al. Cell Metab. 2011, Rohm et al. Cell Metab. 2013), we have tested the response of TBL1 mRNA level to various stimuli. As shown below, TBL1 was indeed upregulated by both cAMP and glucose withdrawal, whereas insulin inhibited TBL1 mRNA levels (Fig. R1), overall suggesting that also in pancreatic tumor cells TBL1 acts as a downstream mediator of endocrine signaling pathways. The further elucidation of these pathways and their functional importance for TBL1-dependent control of tumor cell behavior need to be studied in the future in more detail. We provide the data for the referees only but await their recommendation whether or not to include the data in the actual manuscript.

[Figure omitted from this document upon request by the Authors]

While the data of Fig. 4 are quite convincing (tumor growth and chemo-resistance studies in subcutaneously injected mice), all other results are either incomplete or confusing, as detailed in the point-by point comments here below. In general, to confirm some of the obtained results -that were performed exclusively with silencing of TBL1- at least some of them should be improved i) by using another siRNA and ii) with rescue experiments transferring a TBL1 expression vector which is resistant to the silencing. Moreover, if the authors believe that some of the phenotypes elicited by TBL1 silencing are due to the consequent alteration of glucose uptake or PI3K signalling or both, they should try to demonstrate this by recovering the siTBL1 phenotypes for example by overexpressing PI3K pathway components.

We thank the referee for these valuable comments. Please see our specific responses below.

Major comments:

Fig. 1B is quite misleading for more than one reason: i) first of all normal and tumour tissues are shown with different magnifications (80 versus 25 or 50 micrometers); ii) unequal sections are shown, since the section of normal pancreas (1) derives from an oblique cut of the acini, while in PanIn-1-3 (2-5) longitudinal sections are shown; iii) how many normal and tumour tissues were analysed by IHC?

As outlined in our response to referee #1, a trained pathologist has re-analyzed the data and we have generated a New Fig. 1C. In particular, the magnifications have been harmonized throughout all panels. As the samples are taken and preserved in the surgery hall immediately after resection by the surgeon, we unfortunately have no influence on the embedding orientation in most cases. Still, we feel that these specimens clearly demonstrate the induction of TBL1 expression upon tumor progression. The presented samples are representative out of a total number of 12 samples that were analyzed by IHC.

Fig. 1D: same comment as for Fig. 1B

Please see comment above.

Fig. 2 in general: It seems that only one siRNA sequence was used, or was shRNA sequence different? This is not clear at all. In any case a different sequence for silencing must be added as a control. Moreover, Fig. E3 shows the efficiency of silencing at the mRNA level, but also a Western blot analysis should be added.

As outlined in our response to referee #1, we have repeated key experiments by using additional control and TBL1-specific siRNAs, overall validating our initial findings. In

addition, we have now added a WB and its quantification in a New Fig. E3, showing knockdown of TBL1 on protein level.
Of note, apart from the 2 siRNA sequences directed at human TBL1 for in vitro experiments with human PDAC cell lines, the adenoviral shRNA construct against TBL1 as used in the in vivo experiments carried another, third independent sequence directed at murine TBL1.

Fig. E4B: why in Capan-1 cells also siNC is affecting invasion in a manner similar to siTBL1?

As outlined above, we have also repeated this assay by additional control and TBL1 siRNAs, overall validating the initial conclusions.

Fig. 3C: Explanation of NaCl/GEM or triplicate loading is missing. (One has to arrive at the end of the paper to understand what is GEM). Moreover, some samples, like the first lane of shTBL1/NaCl do not correspond to what was stated in the text, e.g. PTEN and total Akt levels levels are lower than in control cells. Why not showing a representative Western blot and adding a protein density graph with the average {plus minus} st.dev. of all samples? This would be much easier to understand.

We thank the referee for this valuable suggestion. We have changed the order of the figures in the text accordingly. In addition, we have now quantified the WB as shown in a New Fig. E11, demonstrating an almost 2-fold downregulation of PI3K expression upon TBL1 knockdown.

Fig. 3E: ChIP-PCR analysis lacks the control of TBL1 silencing and PCR of an other region in the promoter, different from the peaks in Fig. 3D.

As requested, we have performed additional ChIP studies using TBL1 knockdown cells as well as negative control PCR primers. As outlined in our comments to referee #1, the new data are included in a New Fig. 3C, overall validating specific binding of TBL1 to the PI3K locus.

Minor comments:

Fig. 1C: In the text (pg. 6) related to Fig. 1C, it is not clear why "genome-wide transcriptome analysis" is necessary to cite in this context, since only two genes (TBL1 and PNLIPRP2) were used in this analysis.

We have used an existing transcriptome dataset of a human patient cohort for these analyses and selectively studied TBL1 in this context. PNLIPRP2 was used to normalize the data for tumor-cell content of the specimens.

Fig. 2A, 2B: proliferation defects following TBL1 silencing go from 5% to 30% decrease, depending on species and assay, which is not so much to justify the sentence ".underlining the conclusion that TBL1 fulfills a conserved growth regulatory function..." which is overstated both because of the result and because other experiments should be added to demonstrate this function (e.g. Cell cycle FACS analyses, Western blot of Cell cycle markers etc.).

As requested by the referee, we have softened our statement regarding the function of TBL1 in cellular proliferation. In addition, we have performed FACS analysis of control and TBL1 knockdown cells as shown in a New Fig. E7. In line with the EdU incorporation and cellular proliferation studies, these experiments demonstrated that the inhibition of TBL1 expression leads to a decrease in the number of cells in the S phase while increasing the number of cells in G0/G1.

Again, at the end of the paragraph in pg. 7 the statement ".overall suggesting that TBL1 represents an important checkpoint in the tumorigenic program." is again excessive in view of the confusing results in Fig. E4 and of the type of analyses that have been done till this point that are mainly correlations.

We feel that the referee's comments have further strengthened this manuscript and underlined the role of TBL1 in pancreatic tumor cell biology to justify the above statement. However, we have softened our statement as requested.

Referee #3 (Remarks):

In this paper, the authors show increased expression of TBL1 in human and mouse pancreatic cancers. They correlated TBL1 expression with poor survival in these patients. In vitro and in vivo experiments prove that TBL1 genetic inhibition results in decreased cell proliferation and tumor formation in mice. Finally, the authors show that the effects of TBL1 are mediated by the regulation of the expression of p110 Pi3K in tumor cells. Moreover, the authors claim that the effects of TBL1 are also mediated by the regulation of the metabolic switch in pancreatic cancer cells. The manuscript is well organized, and the experiments are clean. Data is of good quality. This reviewer has, however some major concerns about the mechanisms underlying TBL1 effects. In particular, I have the following concerns:

1. Differences in fig. 2B-C, although significant are very minor and cannot explain major effects on tumor progression.

We agree with the referee that the in vitro effects of TBL1 knockdown are not as impressive as the in vivo consequences of TBL1 inhibition in mouse tumor models. For technical reasons, the cell culture studies only allow a 3 day observation period that is most likely not sufficient to produce stronger effects. In contrast, inhibition of TBL1 in vivo can be achieved over a much longer time period, thus showing more pronounced effects over time.

2. The authors suggest that TBL1 couples proliferative, invasive, and metabolic pathways in pancreatic cancer. This is far to be proved and is only based in a single observation. How is the expression of genes involved in metabolic control changed upon TBL1 inhibition? What are the metabolic effects of TBL1 overexpression in cancer cells? How is glutamine metabolism affected? Is there a major contribution of TBL1 on the metabolic control in normal cells?

We thank the referee for this valuable comment. We have evaluated the effects of TBL1 inhibition on metabolic gene expression (please see New Tab. E1) and found no major effect on metabolic pathway activities. Thus, we conclude that the metabolic changes in terms of glycolysis and glucose uptake capacity upon TBL1 inhibition may represent secondary adaptations to changes in cellular proliferation etc. However, as demonstrated previously by our group (Kulozik Cell Metab. 2011, Rohm Cell Metab. 2013) TBL1 plays a prominent role in lipid metabolism in normal (liver and adipose tissue) cells. Therefore, an in-depth analysis of potential TBL1-dependent effects on tumor cell metabolism seems still justified in future studies. Of note, TBL1 expression also highly significantly correlated with metabolic gene expression in human PDAC, again reflecting either direct or indirect connections in this context.

3. ChIP-seq data analysis is required to understand the whole picture of the participation of TBL1 in these cancer cells. This should be provided. Are there other genes involved in metabolic control that are regulated by TBL1? Or the metabolic effects are also mediated by PI3K? These issues should be addressed.

As outlined above, we did not observe any significant changes in metabolic pathway expression upon TBL1 knockdown, arguing that the metabolic consequences observed may result from secondary adaptations. Thus, we decided to not further explore the metabolic functions of TBL1 in the context of this manuscript so that ChIP-Seq analysis falls beyond the scope of this current manuscript which is focused on the role of TBL1 in cellular proliferation and growth in PDAC.

4. The authors focus on PI3K p110 as a major target of TBL1. Promoter studies showing an effect of TBL1 on PI3K promoter activity would very well complement ChIP data.

As requested by the referee, we have tried to clone the TBL1-targeted region within the PI3K promoter into corresponding reporter vectors. These attempts failed for technical reasons as it remained impossible to amplify the respective genomic regions thus far. However, we feel that

the regulation of endogenous PI3K expression by TBL1 in various models as well as the improved and validated ChIP studies support the identification of PI3K as a TBL1 target in pancreatic cancer cells.

5. The mechanisms of regulation of TBL1 expression are still poorly demonstrated. TBL1 is a transcriptional cofactor that does not have intrinsic transcriptional activity. The question here is: what is the major transcription factor associated to TBL1 in the PI3K promoter? It would very interesting to test if inhibition of this transcription factor would have the same effects on tumor progression.

This is a very interesting point. As shown above (please see Fig. R1), we have observed that TBL1 mRNA levels are under control of cAMP, glucose and insulin signaling pathways in pancreatic tumor cells.
Furthermore, TBL1 as a transcriptional co-activator by definition will interact with numerous DNA-binding factors in different contexts, thus making it unlikely to identify ONE major interaction partner in a certain cellular context. Nevertheless, to further address the referee's comment, we have performed an in-silico analysis of transcription factor binding sites within upstream regions of TBL1-regulated genes. As shown below, TBL1 target genes in pancreatic tumor cells are predominantly enriched in binding sites for Nkx2-5, FOXD3, Elk-1, HLF, NF-Y and GATA-1, suggesting that the TBL1 co-factor function in PDAC also coordinates and wires distinct transcriptional complexes as shown in other cellular contexts. We are providing these data for the referees only but will be happy to include them into the manuscript if advised to do so.

[Table omitted from this document upon request by the Authors]

6. Does PI3K overexpression in pancreatic cancer cells rescue proliferation and tumor formation?

As requested by the referee, we have performed cellular rescue experiments by combining TBL1 siRNA transfection with overexpression of plasmids containing a constitutively active PI3K in HEK cells since Capan-1 cells carry an activating Kras mutation which is known to interact with and activate PI3 kinase. As the cell densities required for either siRNA or plasmid transfection differed substantially, it remained unfortunately impossible to achieve a simultaneous TBL1 knockdown and PI3K overexpression in this system. While we agree on the importance of this question, we ultimately decided to not further pursue these experiments due to time constraints.

2nd Editorial Decision 23 March 2015

Thank you for the submission of your manuscript to EMBO Molecular Medicine. We have now heard back from the three Reviewers, whom we asked to re-evaluate your manuscript.

You will see that while Reviewer 1 is now supportive, Reviewers 2 and 3 are still not satisfied that the issues raised were adequately addressed.

Reviewer 2 essentially reiterates one very important and central issue: the lack of any direct mechanistic evidence that TLB does indeed the cause the phenotypes by up-regulation of the Pi3K/Akt pathway. Reviewer 3, albeit admittedly in a somewhat cursory fashion, does essentially point to the same basic problem. I note that both reviewers did suggest Pi3K over-expression experiments to help address this fundamental issue and I also specifically asked you to address this in my first decision letter. I understand that you had some technical issues in performing these experiments and thus decided to defer, but I am afraid this will not do.

We have now re-discussed your manuscript in the light of these comments and agree that the Reviewers' overlapping concern has merits and would require further, conclusive experimentation for it to be addressed.

As you know, we would normally not allow a second revision. I am prepared in this case, however,

to give you another opportunity to improve your manuscript, with the understanding that the Reviewers' concern must be fully addressed with additional experimentation and that next version of the manuscript will undergo a third and final round of review.

As you know, EMBO Molecular Medicine has a "scooping protection" policy, whereby similar findings that are published by others during review or revision are not a criterion for rejection. However, I do ask you to get in touch with us after three months if you have not completed your revision, to update us on the status. Please also contact us as soon as possible if similar work is published elsewhere.

I look forward to seeing a revised form of your manuscript as soon as possible.

***** Reviewer's comments *****

Referee #1 (Remarks):

The authors have fully addressed my concerns.

Referee #2 (Comments on Novelty/Model System):

As already mentioned in the first review, this manuscript is of great potential for finding new targets against human pancreatic cancer. TLB seems to be at the center between oncogenic signalling and prolifration/metabolism of pancreatic cancer cells, through up-regulation of the PI3K-Akt pathway. Compared to the first submission, the experiments are now also improved in terms of specificity and clarity.

Referee #2 (Remarks):

Regrettably, there is still one missing point, which is of relevance and needs to be addressed: There must be at least one experiment demonstrating that indeed upregulation of the PI3K-Akt pathway by TLB is instrumental for the phenotypes induced by TLB. This means that upon TLB knockdown, PI3K overexpression should rescue for example chemoresistance or glucose influx or invasiveness of pancreatic cancer cells.

Referee #3 (Remarks):

Although some effort has been directed to address reviewers con concerns, unfortunately, after revision, the manuscript has not been improved in the sense that molecular mechanisms are still missing. It remains a paper that correlates expression of TBL1 with cancer.
This reviewer regrets that none of the questions raised in the previous review have been properly addressed by the authors. I cannot therefore accept this new version of the manuscript.

2nd Revision - authors' response 21 April 2015

Referee #1 (Remarks):

The authors have fully addressed my concerns.

We thank the referee for the support.

Referee #2 (Comments on Novelty/Model System):

As already mentioned in the first review, this manuscript is of great potential for finding new targets against human pancreatic cancer. TLB seems to be at the center between oncogenic signalling and

prolifration/metabolism of pancreatic cancer cells, through up-regulation of the PI3K-Akt pathway. Compared to the first submission, the experiments are now also improved in terms of specificity and clarity.

We very much thank the referee for acknowledging the potential and the novelty of our findings.

Regrettably, there is still one missing point, which is of relevance and needs to be addressed: There must be at least one experiment demonstrating that indeed upregulation of the PI3K-Akt pathway by TLB is instrumental for the phenotypes induced by TLB. This means that upon TLB knockdown, PI3K overexpression should rescue for example chemoresistance or glucose influx or invasiveness of pancreatic cancer cells.

This is an important issue. In order to address this point, we have generated murine pancreatic cancer cells stably overexpressing PI3 kinase. These cells were then transfected with TBL1-specific or control siRNA to achieve corresponding control and rescue situations. As shown in our new figures 3D and E, re-constitution of PI3 kinase was indeed able to rescue the impact of TBL1 deficiency on cellular proliferation and invasion. Thus, these data establish PI3 kinase as a functional mediator of the TBL1 pathway in pancreatic cancer cells and support our previous findings on gene expression and chromatin recruitment.

Referee #3 (Remarks):

Although some effort has been directed to address reviewers con concerns, unfortunately, after revision, the manuscript has not been improved in the sense that molecular mechanisms are still missing. It remains a paper that correlates expression of TBL1 with cancer.
This reviewer regrets that none of the questions raised in the previous review have been properly addressed by the authors. I cannot therefore accept this new version of the manuscript.

We strongly disagree with the referee. In particular our newly included data on the functional rescue of TBL1 phenotypes by PI3 kinase re-constitution strongly support our previous findings.

3rd Editorial Decision 28 April 2015

Thank you for the submission of your re-revised manuscript to EMBO Molecular Medicine. We have now received the enclosed reports from the two Reviewers that were asked to re-assess it.

As you will see, while Reviewer 2 is now globally supportive. Reviewer 3 remains quite critical albeit admittedly rather cursory and uninformative.

Considering your rebuttal and that Reviewer 3's initial concerns were partially overlapping with those from Reviewer 2, who is now satisfied, and after in depth discussion with my colleagues, we have decided to provisionally accept your manuscript pending the following amendments/requests to be included in your final revision:

1) As per our Author Guidelines, the description of all reported data that includes statistical testing must state the name of the statistical test used to generate error bars and P values, the number (n) of independent experiments underlying each data point (not replicate measures of one sample), and the actual P value for each test (not merely 'significant' or 'P < 0.05'). I note that you have done so in some, but not all cases (e.g. Figure 4).

2) We are now encouraging the publication of source data, particularly for electrophoretic gels and blots, with the aim of making primary data more accessible and transparent to the reader. Would you be willing to provide a PDF file per figure that contains the original, uncropped and unprocessed

scans of all or at least the key gels used in the manuscript including the supplementary figures? The PDF files should be labeled with the appropriate figure/panel number, and should have molecular weight markers; further annotation may be useful but is not essential. The PDF files will be published online with the article as supplementary "Source Data" files. If you have any questions regarding this just contact me.

3) You are welcome to suggest a striking image or visual abstract to illustrate your article. Should you decide to do so, please provide a jpeg file 550 px-wide x 400-px high.

I look forward to seeing a revised, final form of your manuscript as soon as possible.

***** Reviewer's comments *****

Referee #2:

Is suitable for publication

Referee #3:

Unfortunately nothing changed from my first critics to this manuscript. I am really sorry, but I cannot find any argument to change my decision. My concerns have not been addressed.

3rd Revision - authors' response 13 May 2015

As requested in your provisional acceptance letter, we have now stated the name of the statistical test used to generate error bars and P values, the number (n) of independent experiments underlying each data point, and the actual P value for each test.

We very much hope that these changes are now satisfactory to you.

© EMBO
